# Wheat genetic loci conferring resistance to stripe rust in the face of genetically diverse races of the fungus *Puccinia striiformis* f. sp. *tritici*

**DOI:** 10.1007/s00122-021-03967-z

**Published:** 2021-11-27

**Authors:** Laura Bouvet, Lawrence Percival-Alwyn, Simon Berry, Paul Fenwick, Camila Campos Mantello, Rajiv Sharma, Sarah Holdgate, Ian J. Mackay, James Cockram

**Affiliations:** 1 NIAB, 93 Lawrence Weaver Road, Cambridge, CB3 0LE UK; 2 Department of Plant Sciences, University of Cambridge, Downing Street, Cambridge, CB2 3EA UK; 3 Limagrain UK Ltd, Market Rasen, LN7 6DT UK; 4 Scotland’s Rural College (SRUC), Kings Buildings, West Mains Road, Edinburgh, EH9 3JG UK; 5 Present Address: Scotland’s Rural College (SRUC), The King’s Buildings, West Mains Road, Edinburgh, EH9 3JG UK

## Abstract

**Key message:**

Analysis of a wheat multi-founder population identified 14 yellow rust resistance QTL. For three of the four most significant QTL, haplotype analysis indicated resistance alleles were rare in European wheat.

**Abstract:**

Stripe rust, or yellow rust (YR), is a major fungal disease of wheat (*Triticum aestivum*) caused by *Puccinia striiformis* Westend f. sp. *tritici* (*Pst*). Since 2011, the historically clonal European *Pst* races have been superseded by the rapid incursion of genetically diverse lineages, reducing the resistance of varieties previously showing durable resistance. Identification of sources of genetic resistance to such races is a high priority for wheat breeding. Here we use a wheat eight-founder multi-parent population genotyped with a 90,000 feature single nucleotide polymorphism array to genetically map YR resistance to such new *Pst* races. Genetic analysis of five field trials at three UK sites identified 14 quantitative trait loci (QTL) conferring resistance. Of these, four highly significant loci were consistently identified across all test environments, located on chromosomes 1A (*QYr.niab-1A.1*), 2A (*QYr.niab-2A.1*), 2B (*QYr.niab-2B.1*) and 2D (*QYr.niab-2D.1*), together explaining ~ 50% of the phenotypic variation. Analysis of these four QTL in two-way and three-way combinations showed combinations conferred greater resistance than single QTL, and genetic markers were developed that distinguished resistant and susceptible alleles. Haplotype analysis in a collection of wheat varieties found that the haplotypes associated with YR resistance at three of these four major loci were rare (≤ 7%) in European wheat, highlighting their potential utility for future targeted improvement of disease resistance. Notably, the physical interval for QTL *QYr.niab-2B.1* contained five nucleotide-binding leucine-rich repeat candidate genes with integrated BED domains, of which two corresponded to the cloned resistance genes *Yr7* and *Yr5/YrSp*.

**Graphical abstract:**

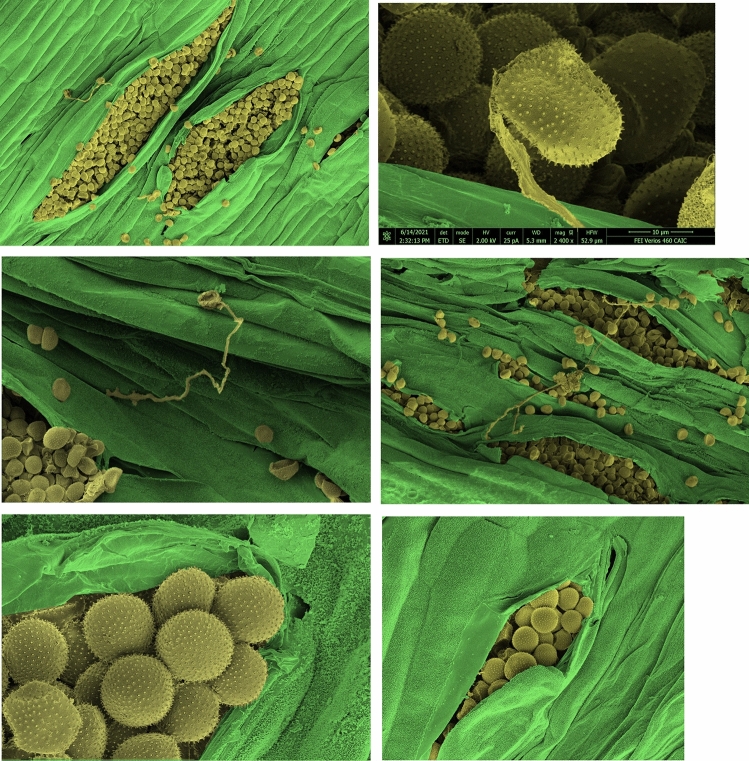

**Supplementary Information:**

The online version contains supplementary material available at 10.1007/s00122-021-03967-z.

## Introduction

Yellow rust (YR), caused by the biotrophic fungus *Puccinia striiformis* Westend f. sp. *tritici* (*Pst*), is a widespread pathogen of wheat (*Triticum aestivum* L.), and a substantial threat to global wheat production. Since the 2000s, a subset of genetically diverse and divergent *Pst* lineages have been responsible for recurrent YR epidemics in numerous wheat-producing regions (Ali et al. 2014, 2017). The rapid adaptation and subsequent spread of these lineages into previously hostile environments has given rise to more aggressive pathotypes that are generally better adapted to higher temperatures (Milus et al. 2009). In the United Kingdom (UK) and North-Western Europe, the historically clonal *Pst* populations have been largely displaced by a genetically diverse group of lineages (Hubbard et al. 2015a, b; Hovmøller et al. 2016). First detected simultaneously in 2011 across several European countries, the ‘Warrior’ and ‘Kranich’ *Pst* races likely originated in the near-Himalayan region and rapidly spread throughout the continent as a group of genetically distinct lineages (Hovmøller et al. 2016). The ability of *Pst* to migrate over long distances, to locally adapt to new environments, and for new lineages to rapidly displace established populations, has had a notable impact on the resistant levels of wheat varieties in the UK (Hubbard et al. 2015a, b; Bueno-Sancho et al. 2017) and beyond (Wellings 2011; Hovmøller et al. 2016), highlighting the importance of the generation of genetic information and resources to support forward YR resistance breeding strategies.

The most efficient way to control the effects of wheat fungal diseases is via approaches that combine agricultural and agronomic practices, disease monitoring and genetic improvement of the wheat varieties grown (Downie et al. 2020). Resistance breeding focuses on two classes of rust resistance (*R*) genes. The first, termed ‘seedling resistance’ or ‘all-stage resistance’, confers qualitative resistance, typically to one or a low number of *Pst* isolates. The second, broadly termed ‘adult plant resistance’ (APR), typically provides quantitative resistance against multiple pathogen races (although APR specificity to *Pst* races does exist, e.g. *Yr12* and *Yr13*. Johnson 1992; McIntosh et al. 1995), and are often effective against multiple biotrophic pathogens (e.g. *Yr18/Lr34/Sr57/Pm38*, conferring resistance to yellow rust, leaf rust, stem rust and powdery mildew; Krattinger et al. 2009). Over 300 genomic regions conferring YR resistance in wheat have been reported (Rosewarne et al. 2013; Wang and Chen 2017). Of these, approximately 80 are permanently designated yellow rust resistance (*Yr*) genes, recently summarised by Jamil et al. (2020). To date, 19 designated *R* genes controlling all-stage resistance to different wheat fungal pathogens have been cloned, and all but two (*Sr60*, a tandem kinase, Chen et al. 2020. *Yr15/YrG303/YrH52*, a kinase-pseudokinase protein, Klymiuk et al. 2018, 2020) encode nucleotide-binding leucine-rich repeat (NLR) proteins: *Lr1* (Cloutier et al. 2007), *Lr10* (Feuillet el al. 2003), *Lr21* (Huang et al. 2003), *Lr22a* (Thind et al. 2017), *Sr13* (Zhang et al. 2017), *Sr21* (Chen et al. 2018), *Sr22* (Steuernagel et al. 2016), *Sr33* (Periyannan et al. 2013), *Sr35* (Saintenac et al. 2013), *Sr45* (Steuernagel et al. 2016), *Sr46* and *SrTA1662* (Arora et al. 2019), *Sr50* (Mago et al. 2015), *Yr5/YrSP*, *Yr7* (Marchal et al. 2018), *Yr10* (Liu et al. 2014; although see also Yuan et al. 2018 who question the result) and *YrAS2388R* (Zhang et al. 2019). Similarly, wheat *R* genes to other fungal pathogens are also encoded by *NLR* genes, such as the powdery mildew resistance genes *Pm2* (Sánchez-Martin et al. 2016), *Pm3b* (Yahiaoui et al. 2004), *Pm8* (Hurni et al. 2013), *Pm21* (He et al. 2018) and *Pm41* (Li et al. 2020). Host NLR proteins predominantly act by recognising the effector molecules that pathogens produce to inhibit host defence responses (Jones et al. 2016). To detect the large range of potential infecting pathogens, plant *NLR* gene families have radiated and diversified, commonly by localised gene duplication, unequal crossing-over and variation within the leucine-rich repeat (LRR) domains which bind pathogen effectors (Sarris et al. 2016). Some *NLRs* have evolved to include additional ‘integrated’ domains that may be involved in receptor activation or downstream signalling (Sarris et al. 2016), most commonly kinase and DNA-binding domains (Andersen et al. 2020; Steuernagel et al. 2020). Examples include the BED zinc finger domain in *Yr7* and two alleles at a closely located paralogous gene termed *Yr5* and *YrSp* (Marchal et al. 2018). NLRs can also act by monitoring the status of host receptor proteins, termed ‘indirect recognition’. For example, the Arabidopsis NLRs RPM1 and RPS2 monitor RPM1-interacting protein 4 (RIN4) for cleavage or phosphorylation by several bacterial effectors (Mackey et al. 2002; Axtell et al. 2003; Andersson et al. 2006). Recently a wheat YR susceptibility gene encoding a branched-chain amino acid aminotransferase, *TaBCAT1*, has been identified via analysis of genes upregulated after *Pst* infection (Corredor-Moreno et al. 2021). In contrast to all-stage resistance genes, each of the three map-based cloned YR adult plant resistance genes encode a different class of protein: *Yr18/Lr34* an ABC transporter (Krattinger et al. 2009) involved in the translocation of abscisic acid (Krattinger et al. 2019), *Yr36* a kinase-START domain protein (Fu et al. 2009) and *Yr46/Lr67* a hexose transporter (Moore et al. 2015). Wheat breeding strategies historically focused on the use of qualitative yellow rust resistance genes, sometimes in isolation. *Yr17* for example was a popular source of such resistance in North-Western Europe. The effectiveness of the genetic resistance conferred by *Yr17* stopped soon after its deployment as a single resistance gene over a large wheat acreage (Bayles et al. 2000). Where possible, resistance breeding strategies now favour the more durable approach of combining race specific and non-race specific resistance genes to provide broad-spectrum resistance (Singh et al. 2005; Chen et al. 2014). Such use of genetic resistance will continue to be aided by the genetic and molecular characterisation of *Yr* genes and quantitative trait loci (QTL), understanding of how these genetic loci interact with each other in inbred and F_1_ genetic backgrounds, availability of molecular markers to track favourable alleles and allelic combinations in breeding programmes, and the development and assessment of resistance gene cassettes containing multiple *R* genes (e.g. Luo et al. 2021).

YR resistance QTL and *Yr* genes have predominantly been identified in biparental (e.g. Rosewarne et al. 2013) and association mapping panels (e.g. Maccaferri et al. 2015; Zegeye et al. 2014; Kertho et al. 2015). The crossing of just two parents in a biparental population inevitably limits the number of resistance genes and alleles that can be investigated (Mackay 2001). While association mapping panels overcome this limitation by exploiting historical recombination events in the germplasm collection used, population structure in such collections can lead to false marker-trait associations, and the effects of rare alleles will not be detected (Cockram & Mackay 2018). Multi-founder populations (reviewed by Cockram & Mackay 2018; Scott et al. 2020) now provide complementary resources for the genetic investigation of wheat disease resistance. Such designs include multiparent advanced generation inter-cross (MAGIC) populations, derived by inter-crossing all founders over multiple generations before the generation of inbred lines. As each MAGIC progeny line likely contains alleles from all founders dispersed throughout its genetic background, MAGIC populations allow the effects of multiple alleles to be assessed within a single unified population. We previously developed the eight-founder ‘NIAB Elite MAGIC’ population, estimated to capture > 80% of the genetic variation observed in UK wheat based on single nucleotide polymorphism (SNP) analysis (Mackay et al. 2014). Here we analyse this MAGIC population for resistance to YR at five trials conducted over two seasons and three sites in the United Kingdom (UK). Eight major adult plant resistance QTL were resolved, identified across growth seasons in which the new genetically diverse ‘Warrior’ group of *Pst* races were endemic in the UK. The gene space of these genetic loci was explored, candidate genes identified and genetic markers tagging resistant haplotypes validated for the four most significant QTL. Additionally, six minor effect QTL were identified. Collectively, this work highlights the role of combinations of adult plant YR resistance genes in the genetic control of the aggressive *Pst* pathotypes that have replaced the previous asexual populations of the pathogen.

## Methods

### Germplasm, trial design and field trials

The ‘NIAB Elite MAGIC’ wheat population (Mackay et al. 2014) consists of eight founders (the winter varieties Alchemy, Brompton, Claire, Hereward, Rialto, Robigus, Soissons and the facultative variety Xi19) inter-crossed over three generations, and the outputs of the crossing then selfed over multiple generations to produce > 1000 recombinant inbred lines (RILs). The population was grown in five field trials over two seasons (YR assessment seasons 2015 and 2016) at three sites in the UK: (1) NIAB-Cambridge trial ground (latitude 52.235010, longitude 0.097871), Osgodby (latitude 53.410161, longitude − 0.386770) and Rothwell (latitude 53.482597, longitude − 0.259779). All trials followed an incomplete randomised block design generated with the DEW experimental design software, formerly www.expdesigns.co.uk but superseded by the R package ‘blocksdesign’ (Edmondson 2020). The numbers of MAGIC RILs and control lines assessed at each site and year combination are listed in Table 1 and Supplementary Table 1. Seed for each season’s trials were sourced from nursery plots grown the preceding season. Accordingly, RIL_9_ and RIL_10_ seed were used for the 2015 and 2016 season trials, respectively. Standard agronomy practices were used for commercial wheat production at each location, but lacking application of chemical protection against YR. All trials were Autumn sown in the calendar year preceding YR assessment. Each line was sown in two 1-m rows, with six rows per plot. At the NIAB trial ground in Cambridgeshire, the central two rows were sown with the spreader wheat variety Vuka, known to be highly susceptible to all known UK races of YR, with RILs on either side. At the Osgodby and Rothwell sites in Lincolnshire, the central two rows were left empty. Instead, Vuka was present as a whole plot every three traverses. All trials apart from OSG16 were inoculated with a mixture of *Pst* races (‘Solstice race’ isolate 08/21 virulent on *Yr 1,2,3,4,6,9,17,25,32* and ‘Warrior race’ isolate 11/08 virulent on *Yr 1,2,3,4,6,7,9,17,25,32,Sp*). Trial OSG16 was un-inoculated, and therefore exposed only to natural YR infection.

**Table 1:** Overview of the five yellow rust MAGIC trials undertaken in the UK during the 2015 and 2016 seasons

Trial	NIAB15	OSG15	ROTH15	NIAB16	OSG16
Location	Cambridge	Osgodby	Rothwell	Cambridge	Osgodby
Total number of plots	2208	1200	1200	1200	1200
Columns × rows	48 cols × 46 rows	20 cols × 60 rows	20 cols × 60 rows	20 cols × 60 rows	20 cols × 60 rows
Main: blocks: subblocks per block	2:46:0	–	–	2:3:2	2:3:2
MAGIC RILs replicated	1085	–	–	444	444
MAGIC RILs unreplicated	–	1060	1060	234	234
MAGIC founders	8	8	8	8^†^	8^†^
Varietal controls	10^a^	2^b^	2^b^	2^†^	2^†^
MAGIC founder controls	–	20^c^	20^c^	–	–
Additional checks	–	Vuka^£^	Vuka^£^	–	–

An unbalanced incomplete random block design was used for all trials

^a^Controls: the yellow rust differential lines Ambition (YR resistance gene *Am*), Cadenza (*Ca*), KWS Sterling (*St*), Rendezvous (*Re*), Solstice (*So*), Spaldings Prolific (*Sp*), Timber (*Ti*), Warrior (*Wa*), the positive control Vuka (–) and the negative control Cougar (*Co*)

^b^Controls: Vuka (positive control) and Cougar (negative control)

^c^20 MAGIC RIL lines used for controls, listed in Supplementary Table 1). All lines and controls were present as two replicates, unless otherwise stated: ^†^Control varieties Oakley (positive control) and Cougar (negative control), each replicated three times. ^£^Susceptible variety Vuka replicated 80 times

### Phenotypic data

YR infection severity of the leaves of adult plants was assessed as the percentage of total leaf tissue with sporulating uredinia, estimated using the modified Cobb’s scale (Peterson et al. 1948) ranging from 0 to 100% infection (%inf). Leaf infection severity was assessed on three to four occasions at 12–18 day intervals at each trial, from the end of booting (Zadoks growth stage 45–49; Zadoks et al. (1974)) until the mid-to-hard-dough stage (growth stage 85–87). Seedling pathotype data for *Pst* isolates collected in 2015 and 2016 by the UK Cereal Pathogen Virulence Survey (UKCPVS) from sites in Cambridgeshire and Lincolnshire (including from Rothwell and Osgodby) were sourced from the relevant annual reports (UKCPVS 2016; UCPVS, 2017). An additional nine isolates collected from our MAGIC trials at NIAB and Rothwell in 2015 were also pathotyped, following protocols described by UKCPVS (2016). The eight MAGIC founders were also assessed for response to four additional UKCPVS *Ptr* isolates belonging to pathotype groups predominant in the test regions in 2015 (Warrior 4 group: 15/057; Warrior 3/Old European group: 15/051) and 2016 (Warrior 4: 16/009; Warrior 1: 16/048), scored using the McNeal scale (McNeal et al. 1971).

### Trials analysis

A stepwise model selection approach was used to estimate the Best Linear Unbiased Estimators (BLUEs) for MAGIC RILs, integrating spatial and non-spatial mixed methods based on Restricted Maximum Likelihood (REML) implemented in Genstat, 18th edition (VSN International 2015). Three models were considered: Model 1—Blocking (genetic effects are estimated based only on the inter- and intra-block variation recovered from the model); Model 2—Spatial (only considers global and/or local field trends); Model 3—Spatial + blocking (combination of the above models). Initially, each model was optimised by including field trends running in either row or column direction or scoring order (the route used to score the trial), followed by between-model comparison to select the one that best fits the data. Further details of the three models used are given in Supplementary Text 1. The Akaike Information Criterion (AIC) was used as a measure for model selection (Akaike 1974). Each model was optimised using AIC as a measure of model fit improvement, and the model with the lowest AIC value selected. Natural log transformation of the disease severity data was performed in cases where residuals were observed not to be normally distributed (trials NIAB16 and OSG16), to improve the normality of the residuals. These were assessed visually with histograms illustrating the distribution of the residuals and Q-Q normality plots. The Shapiro–Wilk normality test was used to further support these observations. Phenotypic correlations were estimated among predicted means using Pearson’s correlation coefficients and paired Wilcoxon signed-rank test using the *Hmisc* package (Harrell 2019). Broad sense heritability (*h*^2^) was used as a measure of total phenotypic variation attributable to the genotypic effect. The VHERITABILITY function in Genstat (18th edition, VSN International 2015) was used to calculate *h*^2^ for each trial and is based on the definition of heritability given by Cullis et al. (2006) and Piepho et al. (2007).

### Genetic analysis

Genetic analysis was carried out using the 7369 SNPs mapped to unique positions on the MAGIC genetic map (Gardner et al. 2016). For all trials, two broad methods were used for genetic analysis, as previously described by Corsi et al. (2020). (1) Single marker analysis (SMA): regression against allelic state at single markers using R/lme4 (Bates et al. 2015) in R (R Core Team 2020) using the following mixed model:

$$Y = \mu_{x} + G_{{\text{m}}} + \beta + e$$

where *Y* is the YR resistance value, *μ* is the adjusted YR score for MAGIC RIL *x*, *G*_m_ is the fixed SNP marker effect, *β* is the population structure consisting of ‘funnels’ and ‘plants within funnels’ effects (from Mackay et al. 2014), and *e* as the residual error term. The model has one degree of freedom, since regression is carried out on binary allelic state. Multiple-test correction was carried out using R/qvalue (Storey 2015), with a threshold of *q* < 0.05. (2) Haplotype-based analysis, for which founder haplotype probabilities were calculated with the mpprob function in R/mpMap (Huang and George 2011) with a threshold of 0.5. Three types of haplotype-based analyses were conducted. Identity by descent (IBD): regression against haplotype probability estimates using R/qtl and the following mixed model:

$$Y = \mu + G_{{\text{p}}} + \beta + e$$

where *Y*, *μ*, *β* and *e* are as for SMA above, and *G*p is the fixed term for founder probabilities. Here, the statistical model has up to seven degrees of freedom. A QTL significance threshold *q* < 0.05 was used. Interval mapping (IM): conducted in R/mpMap using the haplotype probability estimates. Composite interval mapping (CIM): conducted in R/mpMap with 10 covariates using the haplotype probability estimates. Within mpMap, an automated forward selection process based on AIC values was used to select the best ten marker covariates for each MAGIC line. Significant QTLs were then selected in two stages. First, mpMap scans the 100 markers surrounding a particular marker location and selects QTLs based on a threshold of − log10(*p*) > 3. The number of significant QTL were then reduced by fitting a model with *p* < 0.05 and with phenotypic variance explained (PVE) > 0.5%. Significance threshold was estimated based on the simulation of the null distribution. Additionally, R/mpMap outputs founder contributions for each significant QTL, computed using a regression approach at each marker location. To summarise the results of the four analyses, the *p* or *q* values from each mapping method for all adjusted and log transformed YR scores, and for all environments, were compiled into a single table and a ‘consensus’ peak marker for each QTL identified following the methods described in Supplementary Text 2. ‘Major’ and ‘minor’ QTL were defined as those explaining either ≥ 5% or < 5% of the phenotypic variance, respectively.

### Bioinformatic analysis

The locations of the genetically mapped MAGIC SNPs on the physical map were determined using the SNP flanking DNA sequences (Wang et al. 2014) as queries for BLASTn (Altschul et al. 1990) interrogation of the wheat reference genome assembly (RefSeq v1.0, IWGSC 2018). Where BLASTn hits of equal match were identified on more than one chromosome, genetic map position (Gardner et al. 2016) was used to assign BLASTn hits to chromosomes. Cloned wheat rust resistance *R* genes were used as queries for BLASTn searches of the wheat reference genome assembly (RefSeq v1.0) and associated gene annotation (RefSeq v1.1) and significant hits within QTL physical intervals listed. Additionally, analysis of the YR QTL physical intervals in the context of disease resistance gene density, candidate genes were first identified from the IWSGC RefSeq v1.0 assembly (IWSGC, 2018) high- and low-confidence gene functional annotations from v1.0 mapped on to gene model annotation RefSeq v1.1 (Alaux et al. 2018) where transcript.1 contained any of the following search terms: ‘NB-LRR’; ‘NBS-LRR’; ‘NB-ARC’; ‘TIR-NBS’; ‘LRR family protein’; ‘Leucine-rich repeat domain’; ‘Plant disease resistance response’; ‘Nucleotide-binding site leucine-rich repeat’; ‘disease resistance protein (TIR class)’. The percentile ranks of all gene counts and resistant gene counts for each QTL were calculated using windows of the same lengths (9.85–133.91 Mbp) sampling the genome (all 21 chromosomes plus the unassigned chromosome) every 100 bp. Plots were generated using the Circos visualisation tool (Krzywinski et al. 2009). QTL resistant gene enrichment *p* values were calculated using the binomial cumulative probability function where the chance of success is 0.0365 (genome-wide, resistant genes / total genes), the number of tests is the number of genes within the QTL and the number of successes is the number of resistant genes within the QTL.

### Development of KASP markers and haplotype information

Selected SNPs from the 90 k array were converted to the Kompetitive Allele-Specific PCR (KASP) genotyping system. SNP genomic flanking sequences were obtained from the T3 Wheat website (https://triticeaetoolbox.org/wheat/) and used as queries for KASP primer design using Polymarker (Ramirez-Gonzalez et al. 2015) with additional manual curation. The resulting primers (Supplementary Table 2) were used for KASP genotyping as described by Downie et al. (2018), with genomic DNA from the eight MAGIC founders extracted using a DNeasy Plant Mini Kit (Qiagen). To investigate the utility of the KASP markers identified in the MAGIC population against haplotypes at specific loci present in a wider set of germplasm, 403 winter wheat varieties previously genotyped with the 90 k SNP array were parsed from the set of lines available at https://www.niab.com/research/agricultural-crop-research/resources. For each of the four MAGIC QTL investigated, haploblocks were identified in the varietal panel around the location corresponding to the peak of each of the four MAGIC QTLs using Haploview v4.2 (Barrett et al. 2005), allowing the haplotypes they contained to be defined. SNPs able to discriminate between the haplotypes identified in the MAGIC founders and wider panel of 403 varieties were selected for conversion to KASP. For QTL *QYr.niab-2D.1*, difficulties in successfully converting SNPs to KASP meant that a wider window of SNPs was considered.

## Results

### Analysis of YR infection in the MAGIC population

**Fig. 1:**
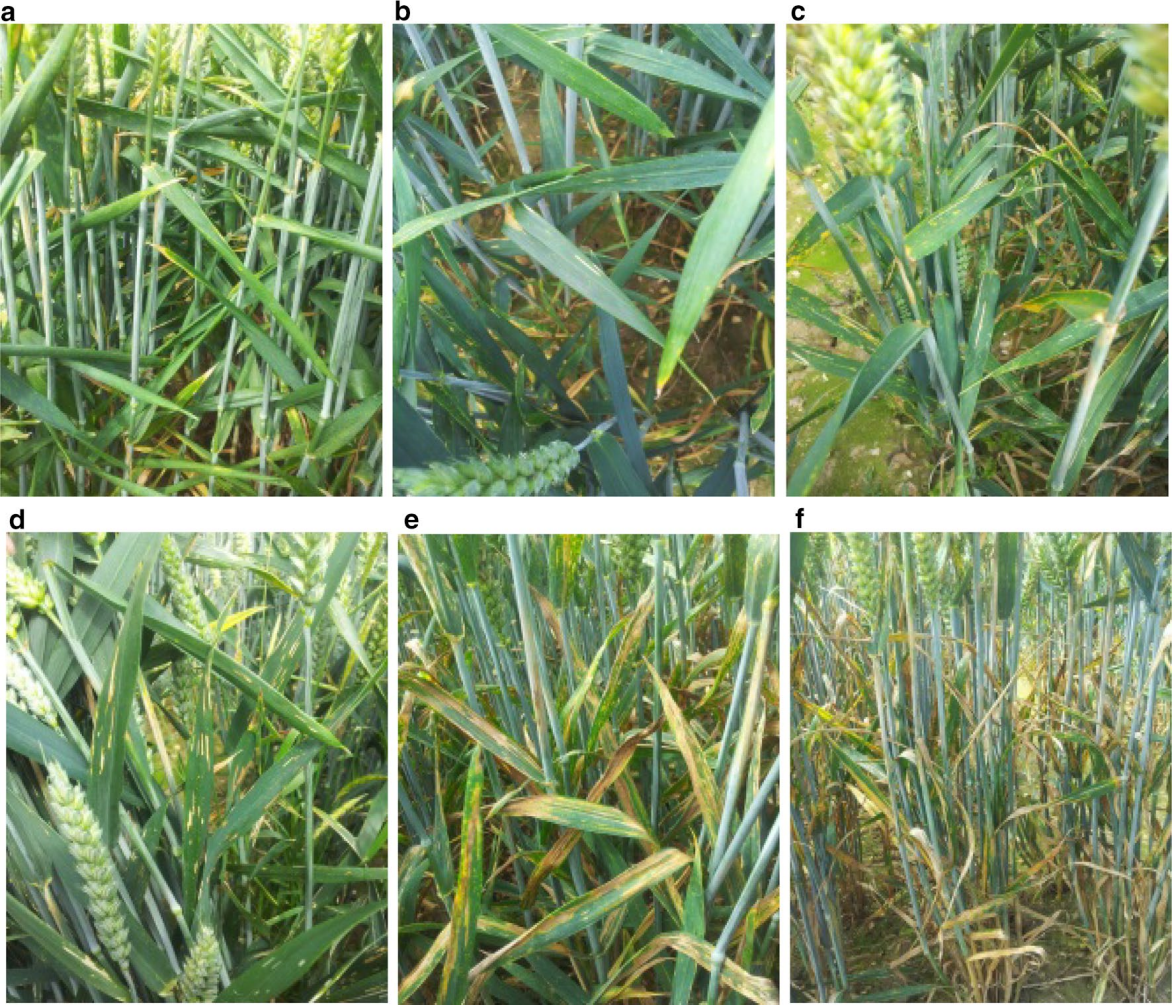
Examples of yellow rust resistance phenotypes in the MAGIC population. Assessments were made using the modified Cobb’s scale (Peterson et al. 1948) ranging from 0 to 100%: **a** score = 0, no infection, 0%, **b** some tillers infected with a few stripes 0–15%, **c** most tillers infected with several stripes, 15–25%, **d** all leaves infected, 25–50%, **e** all leaves infected, 50–75%, **f** little to no green tissue left, > 75% (colour figure online)

To assess YR resistance at the adult plant stage (Fig. 1), infection was assessed in the MAGIC population grown across five trials in the UK, with phenotyping conducted at two time-points per site. Three of the trials were conducted in 2015 (sites NIAB15, OSG15 and ROTH15) and two in 2016 (NIAB16 and OSG16). In 2016, YR scores were skewed towards the resistant end of the scoring scale, and a normal distribution was not observed. Normality for the 2016 season trials was improved using the natural log transformation, and so was used in all subsequent analyses for that year. Normality tests on the 2015 season trials found the residuals to all be normally distributed, and so no further transformation was required. To account for variation within the field, three linear model approaches were applied to the 2015 raw data and the 2016 log transformed data, and the best models selected via Akaike Information Coefficient (AIC) (Supplementary Table 3). YR phenotypic data are listed in Supplementary Table 4.

**Fig. 2:**
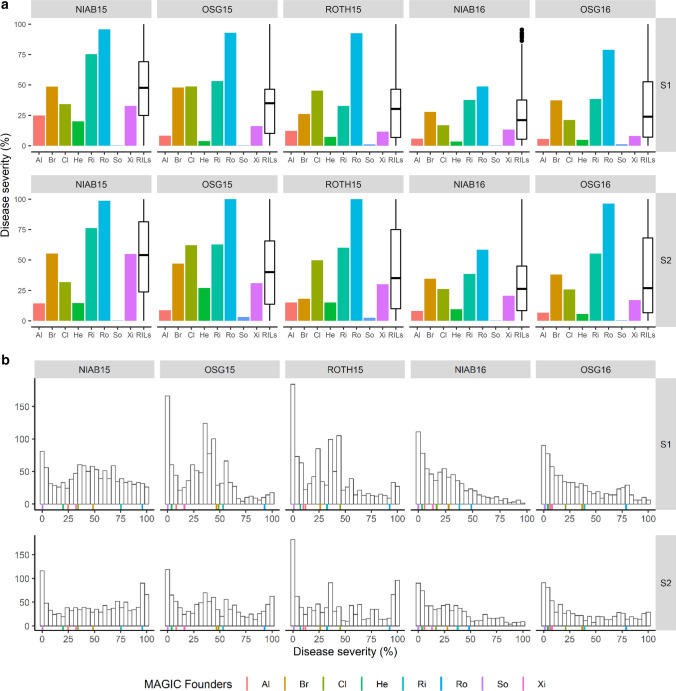
Yellow rust resistance in the MAIGC population. Five test environments: NIAB, Osgodby and Rothwell in 2015 (NIAB15, OSG15, ROTH15) and at NIAB and Osgodby in 2016 (NIAB16, OSG16). At each site, YR infection was recorded at two time-points: S1 (score 1) and S2 (score 2). Al = Alchemy, Br = Brompton, Cl = Claire, He = Hereward, Ri = Rialto, Ro = Robigus, So = Soissons, Xi = Xi19. **a** MAGIC founder percentage YR resistance scores. Additionally, boxplots for all MAGIC recombinant inbred lines are indicated for each trial. **b** Histograms of percentage YR infection in the MAGIC recombinant inbred lines (RILs). Data for the non-transformed adjusted means (BLUEs) are shown for the RILs. BLUEs for each of the eight founders are overlaid below, following the colour coding shown in the key. *Note*: for QTL analysis, the 2016 season data were log transformed. Negative values = 0. Values over 100 = 100 (colour figure online)

Broad sense heritability was high across all test environments (*h*^2^ = 0.93–0.95) (Supplementary Table 3). YR infection scores in the MAGIC founders spanned from 0 to 100%, with similar trends in overall founder ranking observed between sites and years (Fig. 2a). Soissons (0–3% infection) and Robigus (70–100% infection) were the most resistant and susceptible parents, respectively, regardless of year, site and scoring time-point. While the remaining parents varied somewhat in ranking dependent on year and location, two main founder groups were observed: relatively resistant (percentage infection range: 3–33; mean = 10.4: Alchemy, Hereward and Xi19) or relatively susceptible (percentage infection range: 17–76; mean = 36.8: Brompton, Claire and Rialto). The ranking of varieties within a trial remained the same across the two scoring time-points. For the MAGIC RILs, YR scores were similarly distributed at the different sites within each year, but differed considerably between years (Fig. 2b). Nevertheless, correlation of RIL YR scores between test environments was high, ranging from 0.80 to 1.00 (*p* < 0.001). Transgressive segregation in the RILs was observed in 2015 and 2016, both above and below that observed for the most susceptible and resistant founders, respectively (Fig. 2b). In 2015, as YR progressed through the season and susceptibility increased among the MAGIC population, a subset of 31 RILs remained highly resistant (%inf < 1) at all three sites. Intermediate YR scores (10–80%inf) were normally distributed, with this trend more evident at OSG15 and ROTH15, where *Pst* developed more gradually compared to at NIAB15. In 2016, percent YR infection was more skewed towards resistance, compared to the previous year. High levels of resistance (%inf < 1) were maintained by 32 RILs, of which eight were the same as in 2015. Overall, 121 and 171 MAGIC lines exhibited a resistant response (%inf < 10) throughout the 2015 and 2016 scoring seasons, respectively, at all three locations.

### Genetic mapping of yellow rust resistance in MAGIC

**Fig. 3:**
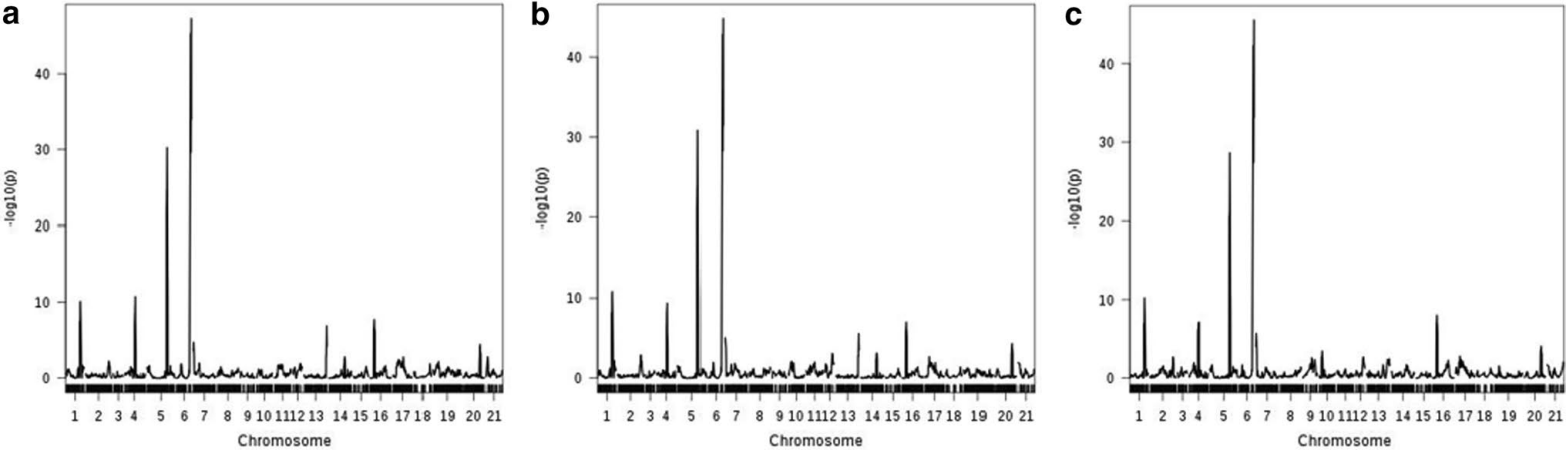
Examples of yellow rust resistance genetic analysis. Shown is the NIAB15 dataset analysed using composite interval mapping with 10 covariates (CIM-cov10), showing QTL scans using the following phenotypes: **a** scoring time-point 1, **b** scoring time-point 1, and **c** scoring time-point 2. All 21 wheat chromosomes are shown, with 1 = chromosome 1A, 2 = 1B, 3 = 1D, through to 21 = 7D

Overall, 14 YR QTL we identified at the adult plant growth stage (Table 2). These could be broadly divided into two groups: eight ‘major’ QTL that explained > 5% of the phenotypic variance, were highly significant (*p* > 1.0E−05) and identified in all trial/score combinations, and six ‘minor’ QTL that explained less than 5% of the variance and had lower significance (*p* < 1.0E−05) (Tables 3, 4). The eight ‘major’ QTL were robustly replicated, identified in all five test environments. While six of these ‘major’ QTL were identified via three or more genetic analysis methods, *QYr.niab-2A.2* and *QYr.niab-6A.2* were identified using IBS and IBD alone. By far the most significant QTL were located on the long arms of chromosomes 2B (*QYr.niab-2B.1*) and 2D (*QYr.niab-2D.1*) (Fig. 3; Table 3), each of which had minimum *p* values < 2.22e^−16^ and explained up to ~ 18% of the phenotypic variance. Predicted allelic effects at *QYr.niab-2B.1* showed Soissons to carry the most resistant allele, while for *QYr.niab-2D.1*, Alchemy and Claire alleles conferred the highest resistance (Table 4). The four ‘major’ QTL on chromosomes 1A, 2A and 3A were found to be the next most significant YR resistance loci, with *p* values ≤ 1.41e^−07^. Of these, the founders Claire and Hereward conferred the strongest resistance at *QYr.niab-1A.1*, Rialto and Xi19 at *QYr.niab-2A.1* and Hereward and Rialto at *QYr.niab-3A.1* (Table 4). As *QYr.niab-2A.2* and *QYr.niab-6A.2* were detected using single marker analysis only, no founder effects were calculated. The two remaining ‘major’ QTL were located on chromosome 6A, had *p* values ≤ 1.22e^−05^ and explained lower amounts (~ 5 to 6%) of the phenotypic variance. At *QYr.niab-6A.1*, the allele(s) from Hereward, Soissons and Xi19 conferred the highest resistance, while for *QYr.niab-6B.1* alleles from Brompton, Rialto and Xi19 conferred resistance.

**Table 2:**
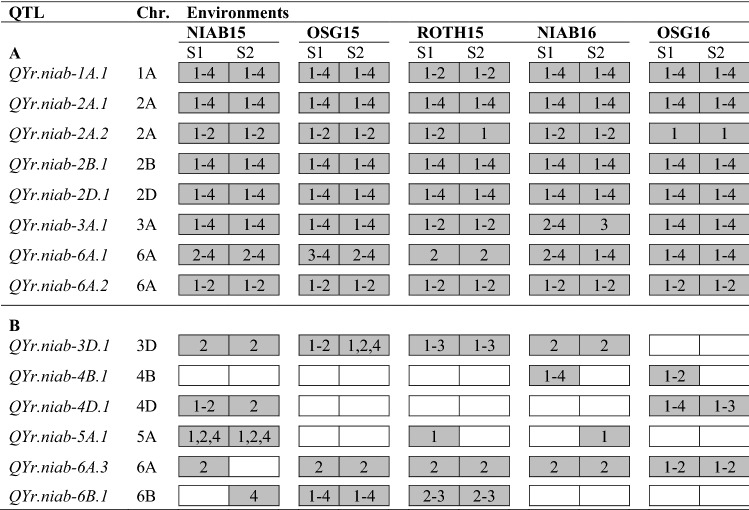
‘Major’ (A) and ‘minor’ (B) yellow rust resistance QTL identified in the MAGIC population using four different QTL analysis methods

QTL analysis methods are numbered as: 1 = single marker analysis, 2 = identity by descent (IBD), 3 = interval mapping, 4 = composite interval mapping with 10 covariates. Trial sites: NIAB, Osgodby (OSG), Rothwell (ROTH). Trial years: 2015 and 2016. Yellow rust resistance phenotyping was undertaken at two time-points: score-1 (S1) and score-2 (S2). Chr. = chromosome. QTL were detected at 5% significance threshold

**Table 3:** Details of the 14 yellow rust (YR) resistance QTL identified in the MAGIC population in seasons 2015 and 2016

QTL	Chr	Peak SNP marker	Genetic position (cM)	Physical position (Mbp)	*p* value		% var. explained		QTL interval (cM)
					Min	Max	Min	Max	
*QYr.niab-1A.1*	1A	*RAC875_rep_c71093_1070*	185.80	568.013	5.72E−10	1.23E−05	7.46	9.26	17.17
*QYr.niab-2A.1*	2A	*BS00022903_51*	140.26	607.827	3.07E−13	1.24E−08	6.80	9.37	19.29
*QYr.niab-2A.2*	2A	*BS00011599_51*	259.39	762.290	1.75E−08	7.91E−03	–	–	2.56
*QYr.niab-2B.1*	2B	*Kukri_c9118_1774*	271.92	683.048	0*	1.58E−11	7.38	17.72	18.62
*QYr.niab-2D.1*	2D	*Ra_c21099_1781*	197.36	638.376	0*	8.62E−12	10.83	18.38	10.85
*QYr.niab-3A.1*	3A	*Kukri_c28650_111*	3.02	7.921	1.41E−07	5.58E−04	3.48	6.02	16.64
*QYr.niab-3D.1*	3D	*BS00004334_51*	162.20	574.773	3.30E−05	1.40E−03	3.35	–	0
*QYr.niab-4B.1*	4B	*Ra_c26080_461*	50.66	36.643	3.72E−05	–	–	2.97	17.73
*QYr.niab-4D.1*	4D	*D_GDRF1KQ02H66WD_341*	125.78	499.107	1.7E−05	2.44E−04	2.89	4.03	26.56
*QYr.niab-5A.1*	5A	*IAAV3916*	301.25	683.343	1.21E−04	9.62E−03	3.48	3.60	13.32
*QYr.niab-6A.1*	6A	*BS00011010_51*	55.51	18.713	4.52E−10	1.22E−05	5.50	6.57	18.24
*QYr.niab-6A.2*	6A	*Kukri_c21743_269*	75.69	27.108	5.15E−10	9.84E−05	–	–	2.05
*QYr.niab-6A.3*	6A	*wsnp_Ex_rep_c101766_87073440*	220.32	596.521	1.49E−04	1.37E−04	–	–	17.96
*QYr.niab-6B.1*	6B	*BS00068615_51*	60.56	54.662	1.44E−07	1.44E−05	2.49	4.08	13.37

The nine ‘major’ QTL are highlighted in bold. Pairs of QTL located in broadly colinear positions on homoeologous chromosomes are underlined. Chr. = chromosome. Genetic map position and QTL interval are from the MAGIC genetic map (Gardner et al. 2016). Minimum (min) and maximum (max) values for *p* value and percentage phenotypic variance explained (% var explained) selected among all environments and QTL mapping methods

* < 2.22E^−16^. QTL interval based on QTL mapping method-4 (composite interval mapping, 10 covariates)

**Table 4:** Predicted MAGIC founder effects at six of the eight ‘major’ yellow rust (YR) resistance QTL

QTL	Chr	Predicted founder effects								Origin (trial, YR score) (trial, YR score)
		Al	Br	Cl	He	Ri	Ro	So	Xi	
*QYr.niab-1A.1*	1A	14.29	7.94	− 3.67	− 12.58	13.31	12.67	4.16	1	NIAB15, S2 (cov0)
*QYr.niab-2A.1*	2A	13.19	19.73	25.82	11.65	3.46	19.59	9.29	1	OSG15, S2 (cov10)
*QYr.niab-2B.1*	2B	− 5.48	0.72	6.79	− 6.41	− 12.6	1.97	− 33.82	1	NIAB15, S2 (cov10)
*QYr.niab-2D.1*	2D	− 21.77	13.46	− 16.70	− 5.12	33.03	21.44	12.13	1	NIAB15, S2 (cov10)
*QYr.niab-3A.1*	3A	1.12	− 4.78	− 6.15	− 16.79	− 22	− 5.16	− 6.81	1	ROTH15, S2 (cov0)
*QYr.niab-6A.1*	6A	16.38	14.57	14.2	7.04	20.69	21.6	7.81	1	NIAB15, S2 (cov10)

Founder effects presented are from the 2015 trials and correspond to the consensus marker with the most significant *p* value identified with QTL mapping methods 2, (identity by descent), 3 (interval mapping, cov = 0) or 4 (composite interval mapping, covariates = 10). For QTL mapping method 2, contributions are relative to Alchemy whereas for methods 3 and 4, they are relative to Xi19. This was calibrated by adding an arbitrary value of 1 to all founder effects. For *QYr.niab-2B.1* and *QYr.niab-2D.1*, founder effects represented are from the peak marker with the highest % variation explained. QTL identified with IBS or IBD analyses only (*QYr.niab-2A.2*, *QYr.niab-6A.2*) are not included

Additionally, six ‘minor’ QTL were identified, located on chromosomes 3D (*QYr.niab-3D.1*), 4B (*QYr.niab-4B.1*), 4D (*QYr.niab-4D.1*), 5A (*QYr.niab-5A.1*), 6A (*QYr.niab-6A.3*) and 6B (*QYr.niab-6B.1*) (Table 3). These each explained a relatively low percentage of the phenotypic variance, ranging from 2.49 to 4.08%. The majority of these ‘minor’ QTL were detected in lower numbers of test environments, although *QYr.niab-6A.3* was detected in all but one site/score combinations analysed. *QYr.niab-4B.1* was only detected in the 2016 season trials and was the only one of the 14 YR QTL identified not to have been replicated between the two seasons.

Pathotype data for 30 *Pst* isolates sampled from the trial sites and surrounding regional areas in the 2015 and 2016 seasons showed the predominant races to be Warrior 3/Old European and Warrior 4 in 2015 and Warrior 4 and Warrior 1 in 2016 (Supplementary Table 5). To support the assumption that these quantitative sources of resistance represented APR mechanisms, rather than all-stage resistance, the eight MAGIC founders were phenotyped for YR infection at the seedling stage to four *Pst* isolates collected in the geographical regions in which the trials were undertaken in 2015 and 2016, and representing the predominant *Pst* races in those regions and years: isolates 15/057 (race Warrior 4), 15/151 (Warrior 3/Old European), 16/009 (Warrior 4) and 16/048 (Warrior 1). Seven of the MAGIC founders were susceptible against all four isolates, while the remaining founder, Xi19, was resistant to one isolate and weakly resistant to the remaining three (Supplementary Table 6). Given that Xi19 was not found to confer resistance alleles at any of the identified loci in the absence of resistance at the same locus from other founders, and that resistance sources were quantitative in nature, our working assumption is that the YR QTL we identified represent APR mechanisms.

### Pairwise QTL interactions

**Fig. 4:**
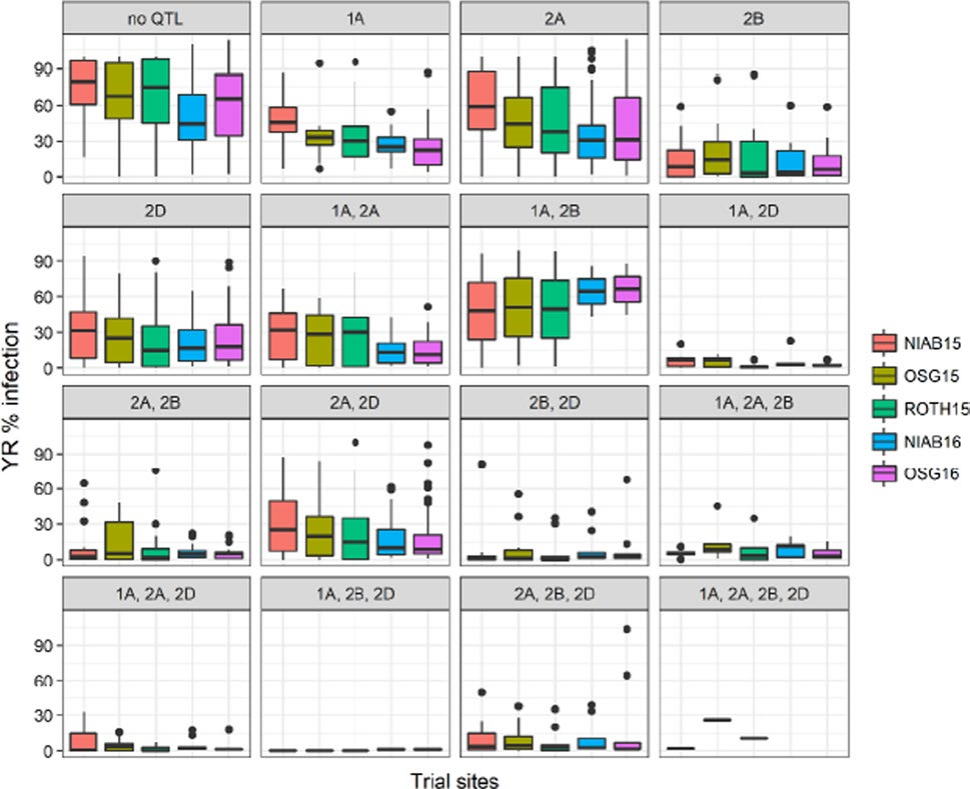
The effects of combining yellow rust (YR) resistance alleles at multiple QTL. Boxplots of the distribution of YR percentage infection in MAGIC recombinant inbred lines (RILs) in each of the five field trials. Based on the presence of resistance alleles at up to four of the most significant MAGIC YR resistance quantitative trait loci (QTL): on chromosomes 1A (*QYr.niab-1A.1*), 2A (*QYr.niab-2A.1*), 2B (*QYr.niab-2B.1*) and 2D (*QYr.niab-2D.1*). Boxes denote the interquartile range, the horizontal lines that bisect them the median, the vertical lines that extend from them the ×1.5 interquartile range beyond it, and the dots the outlier MAGIC RILs

MAGIC RILs were divided into 16 different groups, based on the presence or absence of resistance alleles at the four most significant YR QTLs (*p* < 0.0001, PVE > 8%: *QYr.niab-1A.1, QYr.niab-2A.1, QYr.niab-2B.1* and *QYr.niab-2D.1*) (Fig. 4). For each QTL, calling of the resistant allele was based on the allelic state of the consensus peak markers for each MAGIC founder. A one-way ANOVA found significant differences in the YR disease severity between all the resulting QTL combinations (*p* < 2.2E^−16^, Table 5). This analysis was followed by a pairwise *t* test for the comparison of % disease severity means (Supplementary Table 7). Stacking resistance alleles at all three-way QTL combinations were among the most effective in significantly reducing MAGIC RIL YR infection and displayed high resistance responses to YR infection (*t* test values < 2.0E^−16^). Notably, combining resistance alleles at *QYr.niab-1A.1*, *QYr.niab-2B.1* and *QYr.niab-2D.1* conferred near complete resistance across all trial sites (Fig. 4). Where resistance at *QYr.niab-2D.1* was combined with resistance from *QYr.niab-1A.1* or *QYr.niab-2B.1*, the resulting two-way combinations showed significant reductions in YR susceptibility, comparable to some of the three-way combinations (*t* test values < 2.0E^−16^). All other combinations were also significant in reducing disease severity (4.8E^−16^ < *t* test values > 2.4E^−15^). Single QTL still provided significant levels of resistance against YR but were less effective compared to combinations (*QYr.niab-2D.1*: *t* test value = 1.3E^−13^*, QYr.niab-1A.1*: *t* test value = 4.3E^−10^ and *QYr.niab-2A.1*: *t* test value = 1.8E^−05^). The combination of *QYr.niab-1A.1* and *QYr.niab-2B.1* was the only one found to not significantly reduce disease severity (*t* test value = 0.04). Year and site effects were observed for the QTL combinations *QYr.niab-1A.1*/*QYr.niab-2A.1* and *QYr.niab-1A.1*/*QYr.niab-2B.1*. In both cases, the 2016 trial sites exhibited a narrower range of disease severity response compared to the 2015 sites. Similarly, the *QYr.niab-2A.1/QYr.niab-2B.1* combination was less effective in conferring YR resistance at OSG15 compared to the other trial sites. Notably, the 82 highly YR resistant MAGIC RILs (disease severity < 10%) resistant across all trial sites exhibited all of the 16 QTL combinations investigated, apart from the four-way combination (1A, 2A, 2B, 2D) and *QYr.niab-1A.1* present alone.

**Table 5:** Analysis of variance (ANOVA) in QTL interactions in response to yellow rust disease severity

	SS	df	MS	*F*	*p*
Interactions	27,177.9	15	1811.9	47.2	2.2E^−16^
Residuals	2377.7	62	38.4		

*SS* sum of squares, *Df* degrees of freedom, *MS* mean squares

### Analysis of physical map locations of yellow rust resistance QTL

**Fig. 5:**
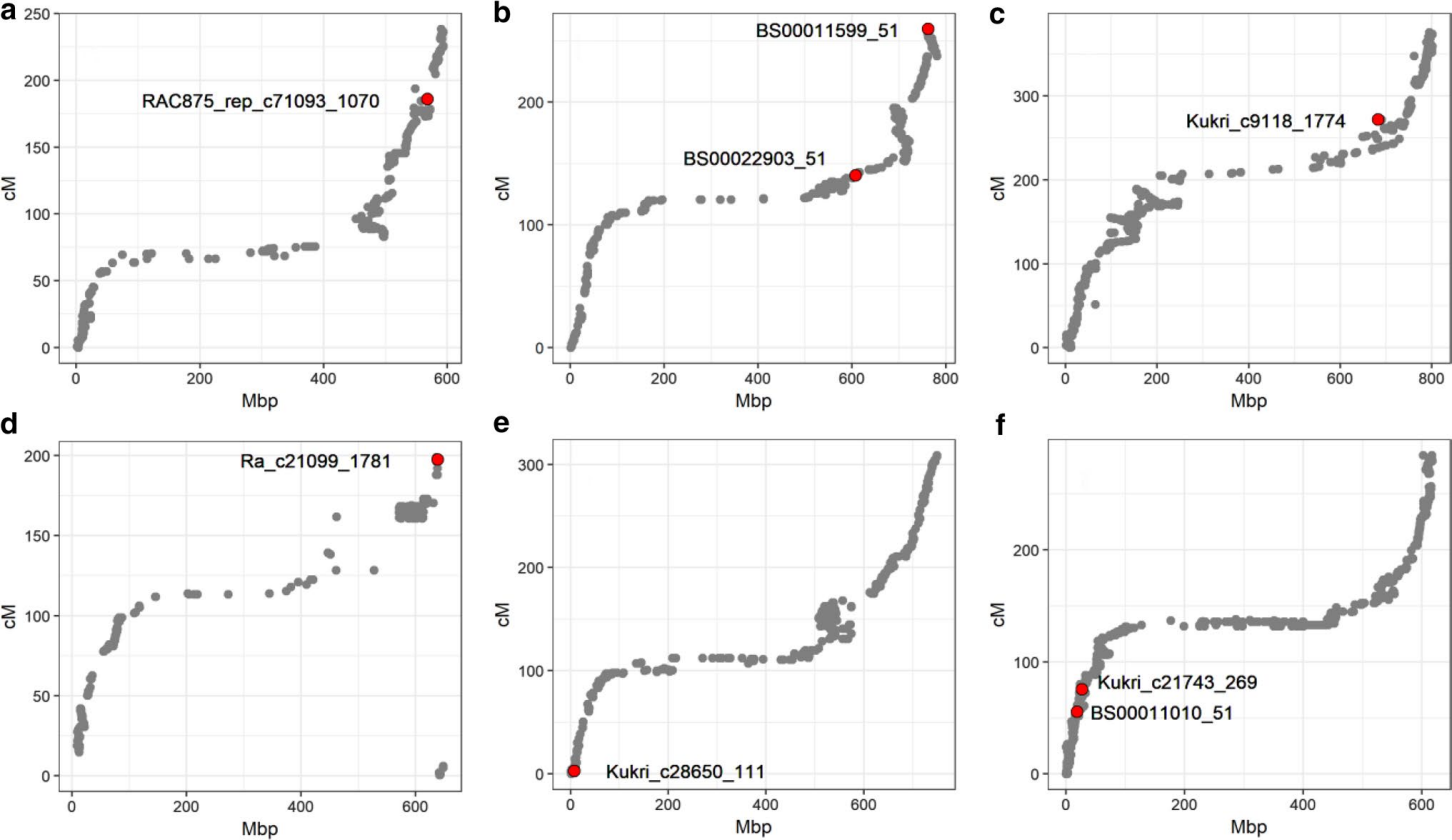
Locations of the eight most significant yellow rust QTL visualised on plots of all genetically mapped SNPs in the MAGIC genetic map (cM) (Gardner et al. 2016) versus their physical map locations (Mbp) (IWGSC Refseq v1.0). Shown are: **a** *QYr.niab-1A.1* (represented by peak SNP *RAC875_rep_c71093_1070*) on chromosome 1A, **b** *QYr.niab-2A.1* (*BS00022903_51*) and *QYr.niab-2A.2* (*BS00011599_51*) on chromosome 2A. **c** *QYr.niab-2B.1*(*Kukri_c9118_1774*) on chromosome 2B. **d** *QYr.niab-2D.1* (*Ra_c21099_1781*) on chromosome 2D. **e** *QYr.niab-3A.1* (*Kukri_c28650_111*) on chromosome 3A. **f** *QYr.niab-6A.1* (*BS00011010_51*) and *QYr.niab-6A.2* (*Kukri_c21743_269*) on chromosome 6A

Anchoring QTL flanking markers to the wheat reference genome found the physical intervals of the eight major and minor YR QTL identified to range from 9.852 Mbp for *QYr.niab-3A.1* to 133.909 Mbp for *QYr.niab-2A.1* (median = 19.839 Mbp) (Supplementary Table 8). To investigate the genomic locations these QTL further, scatterplots of genetic (Gardner et al. 2016) versus physical (IWGSC 2018) map locations were created for the six relevant chromosomes (1A, 2A, 2B, 2D, 3A, 6A), and the locations of the peak SNPs for each QTL highlighted (Fig. 5). While at least half of each of these six chromosomes were located in regions of very low genetic recombination spanning the centromere, all eight major YR resistance QTL were located outside of these regions. Indeed, three of the eight major QTL were located < 18 Mbp from the chromosome telomeres in regions with high genetic recombination (*QYr.niab-2A.2, QYr.niab-2D.1* and *QYr.niab-6A.1*), two were located on the shoulders of the non-recombining regions (*QYr.niab-2A.1*, *QYr.niab-2B.1*), with the remainder located in the intervening regions within which medium levels of genetic recombination typically occur. Of note, four QTL were located in broadly homoeologous (i.e. colinear) genomic locations: the ‘major’ QTL *QYr.niab-2A.2* and *QYr.niab-2D.1* on the long arm of the homoeologous chromosomes 2A and 2D, respectively, and the ‘major’ QTL *QYr.niab-6A.2* was colinear with ‘minor’ QTL *QYr.niab-6B.1* (Supplementary Table 8).

Next, using the wheat reference genome assembly and gene model annotation, we investigated the genomic locations of the 14 YR QTL in respect to overall gene density, and to the density of genes annotation as being involved in disease resistance, including those belonging to the NBS-LRR gene family (Supplementary Fig. 1; Supplementary Table 9). Seven of the 14 QTL were located in genomic regions that contain very high numbers of genes (> 95th percentile, genome-wide). Additionally, four QTL were located in regions with significantly higher number of ‘resistance genes’, based on protein families associated with resistance (> 95th percentile, genome-wide), although these were dominated by NBS-LRR genes typical of all-stage resistance. Furthermore, six QTL had a significantly higher number of ‘resistance genes’ than expected from the QTL gene count, compared to as expected genome-wide, calculated using the binomial cumulative probability function (*p* > 0.05). Details of potential candidate genes within the QTL physical intervals, based on gene model functional annotations and predicted protein domains, as well as sequence searches using the 22 wheat rust *R* genes cloned to date (Supplementary Table 10), are detailed in Supplementary Table 9. In addition to *NLR* genes characteristic of all-stage resistance genes, the colinear *QYr.niab-2A.2* and *QYr.niab-2D.1* were notable for the prominence of receptor-like kinase candidate genes, which encode proteins with protein-kinase and LRR domains. Similarly, the broadly colinear *QYr.niab-6A.2* and *QYr.niab-6B.1* QTL both had high numbers of F-box/LRR domain encoding genes in additional to multiple copies of canonical NLRs, with *QYr.niab-6B.1* also containing a PK-NLR (*TraesCS6B02G099900*; although its 6A homoeologue *TraesCS6A02G093400LC* is a pseudogene and so not annotated as a PK-NLR). *QYr.niab-2B.1* was found to span the wheat reference genome gene models reported to be homologous to the cloned wheat YR resistance genes *Yr7* and *Yr5/YrSP*, represented in the reference genome assembly of cv. Chinese Spring by gene models *TraesCS2B02G488000* and *TraesCS2B02G488600*/*TraesCS2B02G488700*, respectively. Both *Yr7* and *Yr5/SP* encode NLRs with an integrated BED domain, with three additional BED-NLR candidate genes located very close-by (*TraesCS2B02G488400*, *TraesCS2B02G734100LC* and *TraesCS2B02G48900*). With the exception of *Yr7* and *Yr5/YrSP*, none of the remaining 13 cloned wheat *R* genes, or genes with high sequence similarity to these *R* genes, were located in the QTL intervals identified here.

### Development of KASP markers tagging resistant haplotypes

**Fig. 6:**
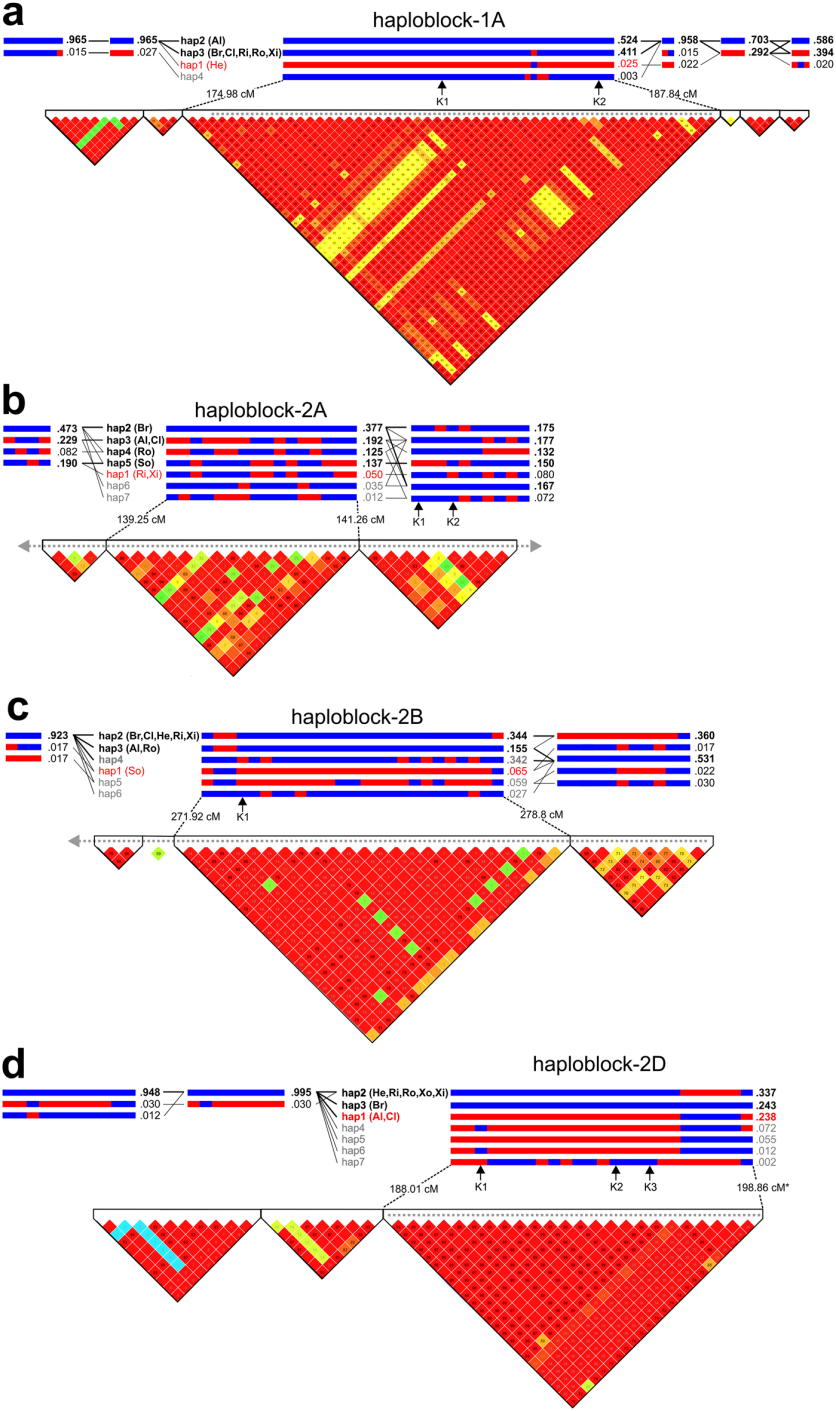
Haplotypes present in a panel of 403 northwest European wheat varieties at each of the four most significant yellow rust QTL identified in the MAGIC population. Haploblocks, and the haplotypes they contain, were determined based on linkage disequilibrium (LD) using genotypic data for the lines derived from the 90 k SNP array. Blue to red colouring indicates increasing LD; values of LD outside of the designated haploblocks are not shown. The haploblock in the varietal panel corresponding to that at the peak of the MAGIC QTL is named (haplpblock-1A, 2A, -2B, -2D), and the extend of the MAGIC QTL interval indicated by the grey dashed line (ending in a grey arrow where the MAGIC QTL intervals extend beyond the haploblocks shown). The frequency of each haplotype in the varietal panel is indicated, with those present at a frequency > 0.10 highlighted in bold. Varietal panel haplotypes containing one or more of the MAGIC founders are indicated as Al (Alchemy), Br (Brompton), Cl (Claire), He (Hereward), Ri (Rialto), Ro (Robigus), So (Soissons), Xi (Xi19), with the haplotype corresponding to the MAGIC founder(s) predicted to confer the highest yellow rust resistance in MAGIC at each locus highlighted in red: **a** *QYr.niab-1A.1* (resistance conferred by the allele from MAGIC founder Hereward), **b** *QYr.niab-2A.1* (Rialto and Xi19), **c** *QYr.niab-2B.1* (Soissons), and **d** *QYr.niab-2D.1* (Alchemy and Claire). The locations of the KASP markers validated for selected SNPs are indicated by the black arrows (colour figure online)

For the four most robust YR QTL investigated above, we used SNPs from the 90 k SNP array able to discriminate resistant and susceptible haplotypes or SNPs in the MAGIC founders and converted these to the KASP genotyping platform (Supplementary Table 2; Supplementary Fig. 2). The utility of these markers was further assessed in terms of ability to discriminate haplotypes within haploblocks identified at the QTL peaks of each of the four loci in a panel of 403 predominantly winter wheat varieties previously genotyped with the 90 k SNP array (Fig. 6; Supplementary Table 11). For MAGIC QTL *QYr.niab-1A.1*, four haplotypes were defined in the variety panel within a large haploblock of 55 SNPs (174.98–187.84 cM) that spanned the majority of the MAGIC *QYr.niab-1A.1* genetic interval (177.50–194.67 cM) (Fig. 6a). Haplotype 1A-hap1 was identified in just 10 varieties (2.5%), including Hereward which was identified as the only MAGIC founder to carry a resistant allele at the QTL. The most common haplotypes, 1A-hap2 (53%, including MAGIC founder Alchemy) and 1A-hap3 (40%, including MAGIC founders Brompton, Claire, Rialto, Robigus and Xi19), differed by just one of the 55 SNPs within the interval, and were both designated susceptible haplotypes, based on the allele effects at this locus identified in the MAGIC population. MAGIC founder Soissons, which carried a susceptible allele at the 1A locus, was one of the 15 varieties suspected to carry a genetic recombination within the haploblock, combining a ‘susceptible’ 1A-hap2/1A-hap3 region with a ‘resistant’ 1A-hap1 region. The co-dominant KASP markers developed for this locus were located on chromosome 1A at 178.013 cM (*BobWhite_c44164_402*) and 185.804 cM (*tplb0021i12_383*), and within this interval discriminated the haplotype associated with resistance (1A-hap1) from the two haplotypes associated with susceptibility (1A-hap2/1A-hap3) (Fig. 6a). The fourth haplotype was only identified in a single variety (Isidor, 1A-hap4). As this haplotype was not captured in the MAGIC founders, it was not possible to predict which class of YR allele it carried.

For *QYr.niab-2A.1*, co-dominant KASP markers were developed for two SNPs (*BS00062679_51* and *BS00022641_51*) which together were able to discriminate the resistant Rialto/Xi19 allele from the more susceptible alleles from the remaining six founders. Analysis of linkage disequilibrium in the variety panel at the peak of the MAGIC QTL identified seven haplotypes, of which four were relatively common (frequency > 10%). These common haplotypes were found in one or more of MAGIC founders carrying susceptible alleles at the locus and were therefore assigned as likely representing susceptible alleles (Fig. 6b). Haplotype 2A-hap1 was present at low frequency in the variety panel (5%), including the two MAGIC founders carrying the resistance allele at the locus: Rialto and Xi19. The two KASP markers (*BS00062679_51* and *BS00022641_51*) were able to discriminate Rialto/Xi19 alleles from the remaining six susceptible MAGIC founders. While analysis in the variety panel found these SNPs to be located in the adjacent haploblock, this was still within the MAGIC QTL interval, and together the two SNPs differentiated the resistant class with all but 12 accessions in the variety panel (Fig. 6). 

For *QYr.niab-2B.1*, analysis of the haploblock in the variety panel corresponding to the location of the peak of the MAGIC QTL identified five haplotypes: 2B-hap1 (6% if varieties, including MAGIC founder Soissons which carried the resistant allele at the locus), 2B-hap2 (34%, including susceptible MAGIC founders Brompton, Claire, Hereward, Rialto, Xi19), 2B-hap3 (16%, including susceptible MAGIC founders Alchemy, Robigus), 2B-Hap4 (34%), as well as the low frequency haplotypes 2B-hap5 (6%) and 2B-hap6 (35). SNP *BS00016650_51* was converted to a co-dominant KASP marker that discriminated the resistant 2B-hap1 haplotype from all but two of the remaining five haplotypes.

Finally, for *QYr.niab-2D.1*, three common haplotypes were identified in the varietal panel: haplotype 2D-hap1 (23% of all varieties, including the MAGIC founders Alchemy and Claire carrying the resistant 2D allele), 2D-hap2 (34%, including MAGIC founders Hereward, Rialto, Robigus, Soissons and Xi19 carrying a susceptible allele), 2D-hap3 (24%, including susceptible MAGIC founder Brompton carrying a susceptible). Four rare haplotypes were also identified, all with a frequency of less than 10% in the variety panel: 2D-hap4 (34%), 2D-hap5 (6%), 2D-hap6 (1%) and 2D-hap7 (the variety, Tyrell). Three KASP markers were developed (*Ra_c19051_1446*, *Kukri_c498_2381*, *RAC875_c50347_258*), which were able to differentiate the resistant haplotype, 2D-hap1, from all remaining haplotypes. However, as the polymorphisms called for all three of these markers were null (unique to the resistant alleles from Alchemy and Claire) versus a SNP call (susceptible alleles from the six remaining founders), each of these three markers assay for presence/absence variation, and are therefore dominant.

## Discussion

### Pst infection at the trial sites

The spread of genetically diverse exotic *Pst* races to European environments and their displacement of the previously clonal *Pst* races has resulted in sudden shifts in wheat resistance ratings. The first of these *Pst* races to be detected in Europe is termed the ‘Warrior’ race (www.wheatrust.org) and was characterised by several notable traits, including relatively large reductions in resistance in varieties that previously carried effective long-term adult plant resistance (Sørensen et al. 2014) and high production of sexual stage spores (teliospores) (Rodriguez-Algaba et al. 2014) indicative of evolution from a sexual population. In 2015, a second genetically diverse *Pst* pathotype called was detected for the first time in the UK, termed the ‘Kranich’ race (UKCPVS 2016). This race is broadly related to the ‘Warrior’ group had been previously detected in continental European countries, and both races are thought to have originated from sexually recombining populations in the near-Himalayan region in Asia (Hovmøller et al. 2016). Four of our trials were inoculated with a mixture of ‘Solstice’ and ‘Warrior’ *Pst* races, while OSG16 was naturally infected. However, natural infection was especially high in both the 2015 and 2016 seasons, with infection pressure being very high even at the time of trial inoculation. Testing of *Pst* isolates from the two locations used in our field trials shows these locations were dominated by natural infection by the Warrior group of races in 2015 and 2016, as was the case within the wider Cambridgeshire and Lincolnshire regions (Supplementary Table 5), and as mirrored across most of the UK (UKCPVS 2012, 2016). Thus, natural infection was most likely predominant in all five trials for the following reasons: (1) the high natural YR infection levels in 2015 and 2016 at these trial sites, as well as across the UK. (2) At our Lincolnshire trial sites OSG and ROTH, which were included in these surveys, the Warrior group of *Pst* races dominated. (3) Our own isolate pathotyping from the NIAB15 and ROTH15 trials. (4) Our observation of natural YR infection prior to trial inoculation, and mirroring regional reports of very high YR disease pressure starting from autumn 2015 when mild conditions allowed rapid early spread (UKCPVS 2012). Therefore, we conclude that the resistance QTL identified in the MAGIC population most likely conferred adult plant resistance to genetically diverse *Pst* races that characterise the recent rapid shifts in the population type of this pathogen that from 2011 replaced the previous clonal *Pst* forms.

### MAGIC yellow rust resistance genes

The YR resistance loci identified at the adult plant stage controlled a large percentage of the phenotypic variance, predominantly accounted for by four loci on chromosomes 1A, 2A, 2B and 2D. Our finding that in almost all cases, combining two or more of these QTL resulted in increased resistance provide experimental evidence that such stacking should be effective in providing strong genetic control for YR. While good resistance was generally provided by stacking two QTL, in practice the use of more loci would provide increased security against future partial or full breakdown of any single resistance locus. While there was no evidence for any of the ‘major’ QTL breaking down over the two seasons investigated, subsequent reports indicate that the resistance conferred by alleles from the MAGIC founders Alchemy and Claire at *QYr.niab-2D.1* has been overcome (Simon Berry, personal communication), indicating it may represent an all-stage resistance locus. Gradual degradation of the effective adult plant YR resistance historically conferred by Claire since its release in 1999 (Powell et al. 2013) can first be traced to the period following the incursion of the ‘Warrior’ group of *Pst* races, when in 2011–2012 Claire went from having the highest resistance score of 9 down to an intermediate score of 6 (UKCPVS 2016). Thus, while Claire’s YR resistance had partially broken down, it nevertheless contained sources of resistance unaffected by at least some of the new *Pst* races present in our 2015 and 2016 seasons, but which was further eroded by the subsequent breakdown of *QYr.niab-2D.1*. Interestingly, four adult plant YR resistance QTL have been identified on chromosomes 2B, 2D and 7B in field-grown trials of a Claire × Lemhi (C × L) population grown in trials between 2003 and 2007 (Powell et al. 2013). The trials were conducted before the introduction of the Warrior type races in 2011, and Claire alleles conferred resistance at both loci. Of these, two QTL co-located with the adult plant resistance loci identified in the MAGIC population. CxL QTL *QYr.niab-2B* overlapped with MAGIC QTL *QYr.niab-2B.1* (based on the CxL physical interval defined by markers *wPt-0950* and *wPt-9190*: 685.047–750.121 Mbp). However, the MAGIC founder Soissons confers the allele with the highest resistance at the *QYr.niab-2B.1* locus, indicating that the underlying loci at these chromosome 2B QTL are most likely different, or that alleles with higher resistance than that conferred by Claire are present. CxL QTL *QYr.niab-2D.2* co-located with our MAGIC QTL *QYr.niab-2D.1* (based on CxL markers *EST18a* and *wmc817a*: 637.651–645.997 Mbp), for which resistance alleles in our MAGIC population were conferred by Claire and Alchemy. Notably, Alchemy has Claire in its pedigree [Alchemy = Clare × (Consort × Woodstock)] (Fradgley et al. 2019). Indeed, the haplotypes of Claire and Alchemy, based on the 90 k SNP data, are identical across the QTL confidence interval (from SNP *RFL_Contig1128_620* to *BS00010685_51*, 188.01–198.86 cM), indicating the chromosome 2D resistance alleles carried by MAGIC founders Claire and Alchemy are identical by descent. The absence of the chromosome 2D and 7B CxL QTL in our MAGIC population may reflect changed virulence profiles of the genetically diverse *Pst* races prevalent in our 2015/2016 season trials compared to the CxL trials that preceded the incursion of exotic *Pst* races. Alternatively, it is possible that none of the MAGIC founders carry the susceptible allele that originated from Lemhi, which is a US variety. Interestingly, a recent study using the ‘NIAB Elite MAGIC’ population found a robust QTL conferring resistance to the necrotrophic fungal pathogen *Parastagonospora nodorum* (the causal agent of Septoria nodorum blotch, SNB) located in the genomic region as the major YR resistance QTL *QYr.niab-2A.1* (Lin et al. 2020). However, comparison of the predicted allelic effects at these QTL finds the resistant alleles at the YR QTL carried by the founders Rialto and Xi19 to be associated with susceptibility for SNB. Analysis of multiple traits in the same population allows such correlations to be identified and further investigated. Indeed, as noted by Scott et al. (2020), the comparatively high genetic diversity and genetic recombination captured by multi-founder populations makes them well suited for the genetic analysis of multiple traits within a single experimental population, so maximising the chances of identifying potential trade-offs between traits.

### Future exploitation of the major YR resistance loci

We identified sources of YR resistance originating from different subsets of the population founders, highlighting the benefit of conducting analyses in multi-founder populations. For the four most significant MAGIC QTL, analysis of the haplotypes present in the varietal panel of 403 accessions indicated between two-to-four ‘common’ haplotypes (defined here as occurring at a frequency of > 10%) were present per locus. The relatively low haplotype diversity at these loci in north-western wheat is in agreement with that identified across the genome in a recent analysis of 16 wheat varieties selected to maximise genetic diversity within this geographic region (Scott et al. 2021). Given such findings, it was perhaps unexpected that for three of our four large effect YR resistance loci, the resistant haplotype was rare in the variety panel (1A-hap1 = 2%, 2A-hap1 = 5%, 2B-hap1 = 7%), further highlighting their possible usefulness as immediate targets for genetic improvement of YR resistance. There could be several reasons for the low frequency of these resistance haplotypes. For example, in the case of the resistance allele conferred by MAGIC founder Hereward at the chromosome 1A QTL, while Hereward was well known and widely grown in the UK for its good grain quality (Mackay et al. 2014), it is the parent of very few subsequent varieties in the wheat pedigree (Fradgley et al. 2019), so reducing the likelihood of further use of the resistance allele in subsequent varieties. Resistance alleles may also be genetically linked to chromosomal regions conferring reduced performance for other agronomically important traits, and so be selected against. Indeed, our chromosome 1A resistance locus was also notable in that the entire QTL interval of ~ 11 cM identified in MAGIC was nested within a wider haploblock of ~ 13 cM in the varietal panel. Given the importance of YR resistance for breeder selection, the rarity of the 1A-hap1 haplotype from Hereward and the relatively large genetic interval of the haploblock in the varietal panel (spanning a chromosomal region that the MAGIC population shows undergoes frequent genetic recombination), it is possible a selective sweep for other agronomically important traits has occurred across this region. Indeed, analysis of changes in allelic diversity across the genome previously identified a SNP adjacent to our peak SNP for the chromosome 1A YR resistance QTL as being under strong selection (located on chromosome 1A at ~ 557 Mbp, based on SNP *BS00032825_51*; Fradgley et al. 2019). Furthermore, genome-wide association scans in a subset of the varietal panel we use here identified a nearby genetic locus controlling grain yield (at ~ 544 Mbp; White et al. 2021). The peak of our chromosome 1A YR resistance QTL lies towards the distal end of the haploblock, at 568 Mbp. As the KASP markers developed here distinguish the resistant 1A-hap1 haplotype from the remaining susceptible haplotypes, these could be used to help combine a distal region containing the YR resistance haplotype 1A-hap1 with a proximal region consisting of the common 1A-hap2 or 1A-hap3 haplotypes, and investigate the consequences on agronomic traits and disease resistance. Thus, consideration of our chromosome 1A YR resistance locus illustrates how complementary sources of genetic, genomic, molecular and phenotypic data and resources might be integrated to effectively exploit sources of genetic resistance.

### Analysis of YR QTL physical intervals

All QTL identified were outside of the pericentromeric regions that are characterised by low genetic recombination and encompass approximately half of the wheat genome (Fig. 5). This, combined with high heritability of the phenotype and the strong contrasting phenotypic effect of the majority of the eight ‘major’ resistance QTL means that it could be useful to exploit the residual heterozygosity in the MAGIC RILs to create near isogenic lines for specific QTL for subsequent fine-mapping studies. Ultimately, combining such knowledge with a better understanding of the genes conferring YR susceptibility, such as the recently identified branched-chain amino acid aminotransferase gene *TaBCAT1* that modulates amino acid metabolism (Corredor-Moreno et al. 2021), as well as gene editing (reviewed by Kumar et al. 2019), may allow future informed design and use of resistance alleles to enhance durable resistance to YR and other wheat pathogens. To date, just three YR adult plant resistance genes have been map-based cloned: *Yr18/Lr34* (Krattinger et al. 2009), *Yr36* (Fu et al. 2009) and *Yr46/Lr67* (Moore et al. 2015), and no homologues of these genes were found within the physical intervals of any of the YR resistance QTL we identified here. However, the all-stage resistance gene *Yr5* is somewhat atypical in that it is known to confer resistance to a broad range of *Pst* isolates worldwide (Marchal et al. 2018). *Yr5* is located in a region of chromosome 2B known to contain numerous yellow rust resistance genes (Feng et al. 2015; Luo et al. 2008), including *Yr7* and *YrSP*. Here we find the physical interval of our MAGIC YR resistance locus *QYr.niab-2B.1* to overlap with the *Yr7* and *Yr5/YrSp* loci. While *Yr7* and *YrSP* no longer provide adequate resistance in the field, *Yr5* continues to remain effective against a range of isolates worldwide (Marchal et al. 2018). *Yr5* (represented in the wheat reference genome of cv. Chinese Spring by gene model *TraesCS2B01G488700*) and *Yr7* (for which no equivalent Chinese Spring gene model is thought to be present; Marchal et al. 2020) are paralogous BED-NLR genes with ~ 78% DNA identity across their coding regions (Marchal et al. 2018). *YrSP* is a truncated allele of *Yr5* resulting in the loss of most of the LRR coding region (Marchal et al. 2018). Resistant *Yr5* and *YrSP* alleles are not reported to be present in European wheat, with diagnostic molecular tests further confirming their absence in all eight MAGIC founders (Marchal et al. 2018). Similarly, molecular analysis has shown *Yr7* to be absent from the MAGIC founders (Marchal et al. 2018). In our MAGIC population, resistance at QTL *QYr.niab-2B.1* came from the Soissons allele. Collectively, this indicates that while *Yr7* and *Yr5/YrSP* do not themselves confer resistance at *QYr.niab-2B.1*, it is possible that different mutations at their underlying genes, or allelic variation at nearby paralogous BED-NLRs in the vicinity, could underly resistance at this locus. Indeed, recent analysis of wheat genome assembly sequences has found copy number variation for BED-NLRs within the vicinity of the *Yr5* and *Yr7* locus (Marchal et al. 2020). Ultimately, further investigation of these, and other, candidates at the QTL is required to narrow down the underlying causative gene and polymorphism. This process will be greatly aided by the finalisation of genome sequence assemblies for all eight MAGIC founders, two of which are currently available (Walkowiak et al. 2020) and the remaining six close to completion, as well as investigation of wheat gene expression resources (e.g. Borrill et al. 2016), including samples collected across different stages of wheat *Pst* infection (Adams et al. 2021).

## Supplementary Information

Below is the link to the electronic supplementary material.

Supplementary file1 (DOCX 14 kb)

Supplementary file2 (XLSX 16 kb)

Supplementary file3 (DOCX 13 kb)

Supplementary file4 (XLSX 201 kb)

Supplementary file5 (DOCX 17 kb)

Supplementary file6 (DOCX 14 kb)

Supplementary file7 (DOCX 19 kb)

Supplementary file8 (DOCX 16 kb)

Supplementary file9 (XLSX 1814 kb)

Supplementary file10 (DOCX 17 kb)

Supplementary file11 (XLSX 4746 kb)

Supplementary file12 (DOCX 15 kb)

Supplementary file13 (DOCX 4394 kb)

Supplementary file14 (DOCX 48 kb)

## Data Availability

Phenotypic data are available in the Supplementary Materials. Germplasm for the ‘NIAB Elite MAGIC’ population is available via https://www.niab.com/research/agricultural-crop-research/resources.
