# Supplementary material for: Wheat genetic loci conferring resistance to stripe rust in the face of genetically diverse races of the fungus Puccinia striiformis f. sp. tritici
Source: Theor Appl Genet. 2021 Nov 27;135(1):301–19. doi: 10.1007/s00122-021-03967-z (PMC8741662; doi:10.1007/s00122-021-03967-z)
Supplement: Supplementary file 1 — Supplementary file1 (DOCX 14 kb) [file 122_2021_3967_MOESM1_ESM.docx]

| **MAGIC RIL** | **Check type** | **2012 nursery YR score^1^** | **2014 nursery YR score^2^** |
| --- | --- | --- | --- |
| MEL_007_2c | Susceptible | 5 | 5 |
| MEL_005_3 | Susceptible | 4 | 5 |
| MEL_018_2 | Susceptible | 5 | 4 |
| MEL_023_4 | Resistant | 2 | 0 |
| MEL_027_1e | Resistant | 1 | 0 |
| MEL_029_2b | Susceptible | 5 | 4 |
| MEL_038_1a | Resistant | 1 | 0 |
| MEL_040_1c | Resistant | 1 | 0 |
| MEL_042_1b | Resistant | 1 | 0 |
| MEL_045_1a | Resistant | 2 | 0 |
| MEL_046_7 | Susceptible | 4 | 4 |
| MEL_052_7 | Susceptible | 4 | 5 |
| MEL_084_4 | Susceptible | 7 | 5 |
| MEL_092_1 | Susceptible | 4 | 4 |
| MEL_093_8 | Resistant | 3 | 1 |
| MEL_104_3 | Susceptible | 4 | 4 |
| MEL_115_3 | Susceptible | 4 | 4 |
| MEL_121_1f | Resistant | 1 | 0 |
| MEL_151_6 | Resistant | 1 | 1 |
| MEL_195_1d | Resistant | 1 | 0 |

**Supplementary Table 1.** NIAB Elite MAGIC recombinant inbred lines (RILs) used as controls for yellow rust (YR) infection at Osgodby 2015 (OSG15) and Rothwell 2015 (ROTH15), selected based on preliminary YR resistance scores phenotyped on 1x1 m un-replicated nursery plots grown in years 2012 and 2014 (P. Howell, unpublished). ^1^Breeders’ resistance scale, scored from 1 (highly resistant) to 9 (highly susceptible). ^2^Scale used: 1 (highly resistant) to 5 (highly susceptible).
