# Supplementary material for: Wheat genetic loci conferring resistance to stripe rust in the face of genetically diverse races of the fungus Puccinia striiformis f. sp. tritici
Source: Theor Appl Genet. 2021 Nov 27;135(1):301–19. doi: 10.1007/s00122-021-03967-z (PMC8741662; doi:10.1007/s00122-021-03967-z)
Supplement: Supplementary file 3 — Supplementary file3 (DOCX 13 kb) [file 122_2021_3967_MOESM3_ESM.docx]

| **Trial** | **Score** | **Best model** | **Broad sense heritability (*h*^2^)** |
| --- | --- | --- | --- |
| NIAB15 | S1 | Blocking + column | 0.94 |
|  | S2 | Blocking | 0.94 |
| OSG15 | S1 | Spatial | 0.95 |
|  | S2 | Spatial | 0.95 |
| ROTH15 | S1 | Spatial | 0.95 |
|  | S2 | Spatial | 0.95 |
| NIAB16 | S1 | Blocking + scoring order | 0.94 |
|  | S2 | Blocking + scoring order | 0.93 |
| OSG16 | S1 | Spatial + scoring order | 0.94 |
|  | S2 | Blocking + spatial + column | 0.95 |

**Supplementary Table 3.** Summary of final models used to correct for field trends for the yellow rust field trials conducted at NIAB, Rothwell (ROTH) and Osgodby (OSG) in seasons 2015 and 2016. Yellow rust severity was scored on two occasions at each trial, denoted as score-1 (S1) and score-2 (S2).
