# Supplementary material for: Wheat genetic loci conferring resistance to stripe rust in the face of genetically diverse races of the fungus Puccinia striiformis f. sp. tritici
Source: Theor Appl Genet. 2021 Nov 27;135(1):301–19. doi: 10.1007/s00122-021-03967-z (PMC8741662; doi:10.1007/s00122-021-03967-z)
Supplement: Supplementary file 5 — Supplementary file5 (DOCX 17 kb) [file 122_2021_3967_MOESM5_ESM.docx]

| **Isolate** | **Location** | **Year** | **New combined pathotype group name** | **Virulence profile** |
| --- | --- | --- | --- | --- |
| 15/186 | Rothwell, Lincolnshire | 2015 | Warrior 3/Old European^1†^ | 1,2,3,4,6,9,17,25,32,Re,Ro,So |
| 15/187 | Rothwell, Lincolnshire | 2015 | Warrior 3/Old European^1†^ | 1,2,3,4,6,9,17,25,32,Re,Ro,So |
| 15/037 | Lincolnshire | 2015 | Warrior 4^1^ | 1,2,3,4,6,7,9,17,25,32,Sp,Ro,So,(Ca), Ap |
| 15/048 | Lincolnshire | 2015 | Warrior 4^1^ | 1,2,3,4,6,7,9,17,25,32,Re,Sp,Ro,So,Ca,St |
| 15/051 | Lincolnshire | 2015 | Warrior 3/Old European^1†^ | ? |
| 15/180 | Lincolnshire | 2015 | Warrior 4^1^ | 1, 2, 3, 4, 6, 7, 9, 17, 25, 32, Sp, Ro, So, Ca, St, Ap |
| 15/057 | Cambridgeshire | 2015 | Warrior 4^1^ | 1,2,3,6,7,9,17,25,32,Sp,Ro,Am,(Ca) |
| 15/058 | Cambridgeshire | 2015 | Warrior 1^1^ | 1,2,3,4,6,7,9,(17),25,32,Sp,Ro,So,Wa,Am,(Ca),Kr |
| NIAB1 | NIAB15 trial site | 2015 | Warrior 3/Old European | 1,3,6,(7),9,(17),(25),(27),(32),Ro,So,(Ca) |
| NIAB2 | NIAB15 trial site | 2015 | Warrior 3/Old European | 1,3,6,7,(9),(17),(25),(27),(32),Ro,(So),Ca |
| NIAB3 | NIAB15 trial site | 2015 | Warrior 4 | 1,3,6,7,9,(17),25,(27),(32),(Sp),Ro,(So),Ca |
| NIAB4 | NIAB15 trial site | 2015 | Warrior 4 | 1,2,3,6,7,9,(17),25,(27),(32),(Sp),(So),Am,(Ca) |
| NIAB5 | NIAB15 trial site | 2015 | Warrior 4 | 1,2,3,(4),6,7,9,(17),(25),(27),(32),(Sp),Ro,(So),Ca |
| NIAB6 | NIAB15 trial site | 2015 | Warrior 4 | 1,2,3,6,7,9,(17),(25),(27),(32),Sp,Ro,Ca,Ti,Ap |
| ROTH1 | ROTH15 trial site | 2015 | Warrior 4 | 1,2,3,4,6,7,9,17,25,27,32,Sp,Ro,So,Ca |
| ROTH2 | ROTH15 trial site | 2015 | Warrior 3/Old European^†^ | 1,2,3,4,6,7,9,17,25,27,32,Rd,Ro,So |
| ROTH3 | ROTH15 trial site | 2015 | Warrior 4 | 1,2,3,5,6,7,9,17,25,27,32,Sp,Ro,(So),(Ca) |
| 16/009 | Cambridgeshire | 2016 | Warrior 4 (Red race 5^2^) | 1,2,3,4,6,7,9,17,25,32,Re,Sp,Ro,So,Ca,Ap |
| 16/048 | Cambridgeshire | 2016 | Warrior 1 (Pink (Path) race 13^2^) | 1,2,3,4,6,7,9,17,25,32,Re,Sp,Ro,So,Wa,Ca,(Ap) |
| 16/199 | Cambridgeshire | 2016 | Warrior 4 (Red race 5^2^) | 1,2,3,4,6,7,9,17,25,32,Re,Sp,Ro,So,Ca,Ap |
| 16/204 | Cambridgeshire | 2016 | Warrior 4 (Red race 11^2^) | 1,2,3,4,6,7,9,17,25,32,Re,Sp,Ro,So,(Wa),Ca,St,Ap |
| 16/205 | Cambridgeshire | 2016 | Warrior 4 (Red race 5^2^) | 1,2,3,4,6,7,9,17,25,32,Re,Sp,Ro,So,Ca,Ap |
| 16/208 | Cambridgeshire | 2016 | Warrior 3 (Blue race 7^2^) | 1,2,3,4,6,7,9,17,25,32,Re,Ro,So,Ev |
| 16/131 | Lincolnshire | 2016 | Warrior 4 (Red race 24^2^) | 1,2,3,4,6,7,9,17,25,32,Re,Sp,Ro,So,Wa,Ca,St,Ap |
| 16/286 | Rothwell, Lincolnshire | 2016 | Warrior 4 (Red race 23^2^) | 1,2,3,4,6,7,9,17,25,32,Re,Sp,Ro,So,Wa,Ca,Ap |
| 16/288 | Rothwell, Lincolnshire | 2016 | Warrior 4 (Red (Path) race 5^2^) | 1,2,3,4,6,7,9,17,25,32,Re,Sp,Ro,So,Ca,(Kr),Ap |
| 16/289 | Rothwell, Lincolnshire | 2016 | Warrior 4 mix (Red race mix^2^) | 1,2,3,4,6,7,8,9,17,25,32,Re,Sp,Ro,So,Wa,Ca,St,Kr,Ap |
| 16/290 | Rothwell, Lincolnshire | 2016 | Warrior 4 (Red race 23^2^) | 1,2,3,4,6,7,9,17,25,32,Re,Sp,Ro,So,Wa,Ca,Ap |
| 16/292 | Rothwell, Lincolnshire | 2016 | Warrior 1 (Pink race 14^2^) | 1,2,3,4,6,7,9,17,25,32,Re,Sp,Ro,So,Wa,Ca,St,Kr,Cr |
| 16/277 | Osgodby, Lincolnshire | 2016 | Warrior 3 (Blue race 10^2^) | 1,2,3,4,6,9,17,25,32,Re,Sp,Ro,So,Wa |

**Supplemental Table 5.** *Pst* pathotypes from samples collected and tested by the UK Pathogen Virulence (UKCPVS) survey from Lincolnshire and Cambridgeshire in 2015 and 2016 These include UKCPVS samples from the Lincolnshire sites at Osgodby and Rothwell included in our MAGIC analysis, as well as samples we collected ourselves from our NIAB15 and ROTH15 MAGIC trials in 2015. Virulence profile corresponds to virulence on yellow rust resistance genes *Yr1, Yr2, Yr3, Yr4, Yr6, Yr7, Yr8, Yr9, Yr17, Yr25, Yr27, Yr32* and on the varieties Rendezvous (Re), Spaldings Prolific (Sp), Robigus (Ro), Solstice (So), Warrior (Wa), Ambition (Am), Cadenza (Ca), KWS Sterling (St), Kranich (Kr), Apache (Ap), Crusoe (Cr), Evolution (Ev) and Timber (Ti). Brackets indicate a borderline virulent response. ^1^UCKVPS 2016. ^2^UKCPVS 2017. ^†^Warrior 3 and Old European races are indistinguishable based on pathotype only, but can be clearly distinguished by genotyping. However, the Old European races have been displaced by Warrior type races in the UK and is no longer seen.
