# Supplementary material for: Wheat genetic loci conferring resistance to stripe rust in the face of genetically diverse races of the fungus Puccinia striiformis f. sp. tritici
Source: Theor Appl Genet. 2021 Nov 27;135(1):301–19. doi: 10.1007/s00122-021-03967-z (PMC8741662; doi:10.1007/s00122-021-03967-z)
Supplement: Supplementary file 6 — Supplementary file6 (DOCX 14 kb) [file 122_2021_3967_MOESM6_ESM.docx]

| ***Pst* isolate (group)** | **Alchemy** | **Brompton** | **Claire** | **Hereward** | **Rialto** | **Robigus** | **Soissons** | **Xi19** |
| --- | --- | --- | --- | --- | --- | --- | --- | --- |
| 15/057 (Warrior 4) | 7.0 | 8.0 | 8.0 | 7.0 | 8.0 | 8.0 | 8.0 | 5.0 |
| 15/151 (Warrior 3/Old European) | 8.0 | 8.0 | 8.0^†^ | 8.0 | 8.0 | 8.0^‡^ | 8.0 | 5.0 |
| 16/009 (Red race 5) | 8.0 | 8.0 | 8.0 | 8.0 | 8.0 | 8.0 | 8.0 | 8.0 |
| 16/048 (Pink race 13) | 8.0 | 8.0 | 8.0 | 8.0 | 8.0 | 8.0 | 8.0 | 6.0 |

**Supplementary Table 6.** Phenotyping the eight MAGIC founder varieties for yellow rust infection at the seedling stage using a 0-9 scale (McNeal et al. 1975), where 0 = immune and 9 = very susceptible, with mean values rounded up or down to the nearest half point on the scale. Infection using isolates sourced from the regions in which the MAGIC YR trials were undertaken in trial years 2015 (isolate 15/057 sourced from Cambridgeshire, 15/151 from Lincolnshire) and 2016 (isolates 16/009 and 16/048 from Cambridgeshire). Host response to infection by each isolate was scored on 10-12 seedlings per variety. ^†^Previously shown to be susceptible at the seedling stage to Warrior 3 isolate 15/151 (UKCPVS, 2016). Access to all isolates was provided by the UK Cereal Pathogen Virulence Survey (UKCPVS) at NIAB.
