# Supplementary material for: Wheat genetic loci conferring resistance to stripe rust in the face of genetically diverse races of the fungus Puccinia striiformis f. sp. tritici
Source: Theor Appl Genet. 2021 Nov 27;135(1):301–19. doi: 10.1007/s00122-021-03967-z (PMC8741662; doi:10.1007/s00122-021-03967-z)
Supplement: Supplementary file 7 — Supplementary file7 (DOCX 19 kb) [file 122_2021_3967_MOESM7_ESM.docx]

|  | **1a** | **1a_2a** | **1a_2a_2b** | **1a_2a_2b_2d** | **1a_2a_2d** | **1a_2b** | **1a_2b_2d** | **1a_2d** | **2a** | **2a_2b** | **2a_2b_2d** | **2a_2d** | **2b** | **2b_2d** | **2d** |
| --- | --- | --- | --- | --- | --- | --- | --- | --- | --- | --- | --- | --- | --- | --- | --- |
| **1a_2a** | 0.0039 | - | - | - | - | - | - | - | - | - | - | - | - | - | - |
| **1a_2a_2b** | 2.0E^-08^ | 0.0012 | - | - | - | - | - | - | - | - | - | - | - | - | - |
| **1a_2a_2b_2d** | 1.3E^-05^ | 0.0359 | 0.4087 | - | - | - | - | - | - | - | - | - | - | - | - |
| **1a_2a_2d** | 3.2E^-10^ | 3.4E^-05^ | 0.3043 | 0.0888 | - | - | - | - | - | - | - | - | - | - | - |
| **1a_2b** | 6.7E^-07^ | 4.7E^-12^ | <2E^-16^ | 9.0E^-14^ | <2E^-16^ | - | - | - | - | - | - | - | - | - | - |
| **1a_2b_2d** | 3.5E^-12^ | 5.0E^-07^ | 0.034 | 8.6E^-03^ | 0.2597 | <2E^-16^ | - | - | - | - | - | - | - | - | - |
| **1a_2d** | 1.9E^-10^ | 2.1E^-05^ | 0.2481 | 7.0E^-02^ | 0.8968 | <2E^-16^ | 0.3178 | - | - | - | - | - | - | - | - |
| **2a** | 6.1E^-03^ | 2.0E^-07^ | 2.5E^-13^ | 9.5E^-10^ | 4.4E^-15^ | 0.0091 | <2E^-16^ | 2.7E^-15^ | - | - | - | - | - | - | - |
| **2a_2b** | 1.0E^-08^ | 6.2E^-04^ | 8.7E^-01^ | 3.3E^-01^ | 3.9E^-01^ | <2E^-16^ | 0.0495 | 3.2E^-01^ | 1.3E^-13^ | - | - | - | - | - | - |
| **2a_2b_2d** | 5.0E^-07^ | 1.1E^-02^ | 4.1E^-01^ | 9.1E^-01^ | 6.7E^-02^ | <2E^-16^ | 0.0038 | 5.1E^-02^ | 6.5E^-12^ | 0.3220 | - | - | - | - | - |
| **2a_2d** | 1.9E^-03^ | 8.1E^-01^ | 2.2E^-03^ | 5.8E^-02^ | 7.9E^-05^ | 1.8E^-12^ | 1.3E^-06^ | 5.0E^-05^ | 7.9E^-08^ | 0.0013 | 0.0212 | - | - | - | - |
| **2b** | 1.3E^-05^ | 8.6E^-02^ | 9.6E^-02^ | 5.3E^-01^ | 8.3E^-03^ | 5.0E^-15^ | 2.7E^-04^ | 5.8E^-03^ | 2.0E^-10^ | 0.0676 | 0.3922 | 0.1383 | - | - | - |
| **2b_2d** | 2.2E^-08^ | 1.2E^-03^ | 9.8E^-01^ | 4.2E^-01^ | 2.9E^-01^ | <2E^-16^ | 3.2E^-02^ | 2.4E^-01^ | 2.7E^-13^ | 0.8439 | 0.4264 | 0.0024 | 0.1015 | - | - |
| **2d** | 3.7E^-02^ | 3.9E^-01^ | 6.0E^-05^ | 5.2E^-03^ | 1.4E^-06^ | 1.5E^-10^ | 1.7E^-08^ | 8.5E^-07^ | 5.5E^-06^ | 3.3E^-05^ | 0.0009 | 0.2693 | 0.0112 | 0.0001 | - |
| **no_QTL** | 1.2E^-10^ | 9.0E^-16^ | <2E^-16^ | <2E^-16^ | <2E^-16^ | 3.2E^-02^ | <2E^-16^ | <2E^-16^ | 7.5E^-06^ | <2E^-16^ | <2E^-16^ | 3.6E^-16^ | <2E^-16^ | <2E^-16^ | 2.5E^-14^ |

**Supplementary Table 7**. QTL interactions between the four most significant yellow rust QTLs, located on chromosomes 1A (*QYr.niab-1A.1*)*,* 2A (*QYr.niab-2A.1*)*,* 2B (*QYr.niab-2B.1*) and 2D (*QYr.niab-2D.1*). *P-*value outputs from pairwise comparisons of QTL combinations with *t*-tests are shown. Significance threshold used = *p*<0.01. Columns and row headings represent the different QTL combinations e.g. 1a_2a represents MAGIC RILs with yellow rust resistance alleles at both *QYr.niab-1A.1* and *QYr.niab-2A.1*.
