# Supplementary material for: Wheat genetic loci conferring resistance to stripe rust in the face of genetically diverse races of the fungus Puccinia striiformis f. sp. tritici
Source: Theor Appl Genet. 2021 Nov 27;135(1):301–19. doi: 10.1007/s00122-021-03967-z (PMC8741662; doi:10.1007/s00122-021-03967-z)
Supplement: Supplementary file 8 — Supplementary file8 (DOCX 16 kb) [file 122_2021_3967_MOESM8_ESM.docx]

| **QTL^1^** | **Chr.** | **Peak SNP position (bp)^2^** | **Start (bp)^2^** | **Stop (bp)^2^** | **Interval (Mbp)^2^** | **Genes^3^** | **Gene percentile rank^4^** | **Resistance genes^5^** | **Resistance gene percentile rank^6^** | ***P*-value^7^** |
| --- | --- | --- | --- | --- | --- | --- | --- | --- | --- | --- |
| ***QYr.niab-1A.1*** | 1A | 568,012,820 | 545888499 | 577841123 | 31.953 | 1328 | **100** | 41 | 88 | 0.848 |
| ***QYr.niab-2A.1*** | 2A | 607,827,264 | 543620284 | 677529736 | 133.909 | 2214 | 63 | 57 | 66 | 0.997 |
| ***QYr.niab-2A.2*** | 2A | 762,290,086 | 761248623 | 780798557 | 19.550 | 830 | **99** | 49 | **97** | **0.001** |
| ***QYr.niab-2B.1*** | 2B | 683,047,583 | 635023684 | 748985682 | 113.962 | 2630 | 85 | 95 | 86 | 0.517 |
| ***QYr.niab-2D.1*** | 2D | 638,376,433 | 637636368 | 651852609 | 14.216 | 689 | **100** | 71 | **99** | **0.000** |
| ***QYr.niab-3A.1*** | 3A | 7,920,709 | 1 | 9852685 | 9.853 | 286 | 86 | 14 | 88 | 0.105 |
| *QYr.niab-3D.1* | 3D | 574,773,288 | 536029042 | 596708808 | 60.680 | 2009 | **97** | 102 | **96** | **0.001** |
| *QYr.niab-4B.1* | 4B | 36,642,747 | 28716585 | 132334233 | 103.618 | 1732 | 63 | 43 | 63 | 0.996 |
| *QYr.niab-4D.1* | 4D | 499,108,143 | 482705858 | 509857067 | 27.151 | 1018 | **97** | 34 | 88 | 0.665 |
| *QYr.niab-5A.1* | 5A | 683,342,769 | 682933090 | 709773743 | 26.841 | 857 | 92 | 23 | 80 | 0.927 |
| ***QYr.niab-6A.1*** | 6A | 18,713,189 | 10493377 | 23434335 | 12.941 | 538 | **98** | 47 | **98** | **0.000** |
| ***QYr.niab-6A.2*** | 6A | 27,108,470 | 26977185 | 47106025 | 20.129 | 595 | 88 | 36 | 93 | **0.001** |
| *QYr.niab-6A.3* | 6A | 596,521,256 | 592818078 | 607956733 | 15.139 | 620 | **98** | 25 | 91 | 0.264 |
| *QYr.niab-6B.1* | 6B | 54,662,261 | 41706023 | 121489184 | 79.783 | 1668 | 77 | 77 | 87 | **0.018** |

**Supplementary Table 8.** Analysis of physical intervals and gene content within for the 14 yellow rust resistance quantitative trait loci (QTL). ^1^‘Major’ YR resistance QTL (each explaining >5% of the phenotypic variance) are highlighted in bold. ^2^Based on the wheat reference genome (RefSeq v1.0; IWGSC, 2018). ^3^Gene model build: RefSeq v1.1). ^4^Calculated using an interval the size of each target QTL, sampling the genome every 100 bp. ^5^Number of resistance genes, as defined in the Methods section. ^6^Number of resistance genes, as defined in the Methods section. ^7^‘Resistance gene’ enrichment value (binomial cumulative probability).
