# Supplementary material for: Wheat genetic loci conferring resistance to stripe rust in the face of genetically diverse races of the fungus Puccinia striiformis f. sp. tritici
Source: Theor Appl Genet. 2021 Nov 27;135(1):301–19. doi: 10.1007/s00122-021-03967-z (PMC8741662; doi:10.1007/s00122-021-03967-z)
Supplement: Supplementary file 10 — Supplementary file10 (DOCX 17 kb) [file 122_2021_3967_MOESM10_ESM.docx]

| **Cloned wheat rust resistance gene** | ***R* gene class** | **NCBI protein accession number** | **Gene functional annotation** | **Reference** |
| --- | --- | --- | --- | --- |
| *Lr1* | ASR | ABS29034 | CC-NBS-LRR | Cloutier et al. 2007 |
| *Lr10* | ASR | AAQ01784 | CC-NBS-LRR | Feuillet et al. 2003 |
| *Lr21* | ASR | ACO53397 | NBS-LRR | Huang et al. 2003 |
| *Lr22a* | ASR | ARO38244 | CC-NBS-LRR | Thind et al. 2017 |
| *Sr13* | ASR | ATE88995 | CC-NBS-LRR | Zhang et al. 2017 |
| *Sr21* | ASR | AVK42833 | CC-NBS-LRR | Chen et al. 2018 |
| *Sr22* | ASR | CUM44200 | CC-NBS-LRR | Steuernagel et al. 2016 |
| *Sr33* | ASR | AGQ17384 | CC-NBS-LRR | Periyannan et al. 2013 |
| *Sr35* | ASR | AGP75918 | CC-NBS-LRR | Saintenac et al. 2013 |
| *Sr45* | ASR | CUM44213 | CC-NBS-LRR | Steuernagel et al. 2016 |
| *Sr46* | ASR | AYV61514 | CC-NBS-LRR | Arora et al. 2019 |
| *Sr50* | ASR | ALO61074 | CC-NBS-LRR | Mago et al. 2015 |
| *Sr60* | ASR | LRRK123 | Tandem kinase | Chen et al. 2020 |
| *SrTA1662* | ASR | *Not listed* | CC-NBS-LRR | Arora et al. 2019 |
| *YrAS2388* | ASR | QDW65446 | CC-NBS-LRR | Zhang et al. 2019 |
| *Yr5/YrSP* | ASR | QEQ12705/QEQ12706 | BED-NBS-LRR | Marchal et al. 2018 |
| *Yr7* | ASR | QEQ12704 | BED-NBS-LRR | Marchal et al. 2018 |
| *Yr10^*^* | ASR | AAG42168 | CC-NBS-LRR | Liu et al. 2014 |
| *Yr15/YrG303/YrH52* | APR | AXC33067 | TKP | Klymiuk et al. 2018 |
| *Yr18/Lr34* | APR | ACN41354 | ABC transporter | Krattinger et al. 2009 |
| *Yr36* | ASR | ACF33187 | Kinase-START | Fu et al. 2009 |
| *Yr46/Lr67* | ASR | ALL26331 | Hexose transporter | Moore et al. 2015 |

**Supplementary Table 10.** Cloned wheat rust resistance *R* gene-encoding protein sequences used as queries for tBLASTn search against the *Triticum aestivum* RefSeq v1.0 genome (IWGSC, 2018) and associated gene model annotation (RefSeq v1.1). ASR = all-sage resistance. APR = adult plant resistance. ^*^See Yuan et al. (2018), who state the CC-NBS-LRR gene identified by Liu et al. (2014) may not represent *Yr10*.
