# Supplementary material for: Wheat genetic loci conferring resistance to stripe rust in the face of genetically diverse races of the fungus Puccinia striiformis f. sp. tritici
Source: Theor Appl Genet. 2021 Nov 27;135(1):301–19. doi: 10.1007/s00122-021-03967-z (PMC8741662; doi:10.1007/s00122-021-03967-z)
Supplement: Supplementary file 12 — Supplementary file12 (DOCX 15 kb) [file 122_2021_3967_MOESM12_ESM.docx]

**Supplementary Text 1.** Details of the models used for trials analysis.

Model 1: Blocking. Used a baseline model consisting of a fixed genotypic effect for MAGIC lines, a random block effect and an error term. The block effect consisted of different terms depending on the trial: ‘block’ for trial NIAB15, ‘rep/block’ for OSG15 and ROTH15 and ‘rep/block/sub_block’ for NIAB16 and OSG16. The ‘/’ operator denotes nested terms. Model 1 can be formulated as:

$y=\mathbf{X}_{g}g+\mathbf{Z}u+e$

where $y$ is the vector of yellow rust (YR) infection in a column x row matrix, $\mathbf{X}_{\boldsymbol{g}}g$ contains the fixed effect for MAGIC lines, $\mathbf{Z}u$ contains the random block effects and $e$ is the residual error term. Model 1 was optimised by independently fitting random terms for column and ScO.

Model 2: Spatial. MAGIC lines were also included as fixed effects. Global trends were incorporated as trends aligned with rows and/or columns. Local trends were incorporated in the model with correlated residuals from the first-order autoregressive processes across rows and columns, now referred to as the AR1 x AR1 model. Model 2 can be formulated as:

$y=\mathbf{X}_{g}g+\mathbf{X}_{\beta}\beta+\xi+e$

where $\mathbf{X}_{g}g$ contains the same fixed terms as Model 1 and vector $\beta$ and $\xi$ contain the spatial trends. Vector $\beta$ may include linear global trends while $\xi$ contains the local trends as the spatially correlated residuals from the AR1 x AR1 model. $e$ is the residual error term. Model 2 was optimised by fitting global trends and/or an additional random term for ScO.

Model 3: Blocking + Spatial. The non-spatial model serves as a baseline to the addition of spatial trend terms, following the sequential approach used by Gilmour et al. (1997). This combined model can be formulated as:

$y=\mathbf{X}_{g}g+\mathbf{X}_{\beta}\beta+\mathbf{Z}u+\xi+e$

where $\mathbf{X}_{g}g$, $\mathbf{X}_{\beta}\beta$, $\mathbf{Z}u$, $\xi$ and $e$ are defined as in model formulations (4) and (5).

**Supplementary Text 2.** Methodology used to determine a ‘consensus’ peak SNP at each QTL based on the four types of genetic analyses undertaken.

Firstly, the most significant marker at each QTL interval was manually selected for SMA and IBD approaches. These locations were then compared to those identified for the IM and CIM approaches using R/mpMap and ‘consensus’ overall peak markers were selected using the following criteria:

- Consensus between the different mapping approaches: for each QTL mapping approaches, the peak marker at a given QTL interval was the marker with the most significant p and q values. This value was compared across all mapping approaches. Markers found significant only with CIM 10 covariates were not considered.
- Consensus between the different YR scores across 2015 and 2016: A marker was selected as an overall peak marker at a given QTL interval when it was the most significant in at least two YR scores in a trial.

In addition, the genomic context (centromere, introgressions, translocations) and marker density were also taken into consideration when selecting an overall peak marker.

A single peak marker was then identified by examining the physical map position of each peak marker candidate in the reference wheat genome generated from cv. Chinese Spring 42 (RefSeq v1.0; IWGSC 2018).
