# Supplementary material for: Wheat genetic loci conferring resistance to stripe rust in the face of genetically diverse races of the fungus Puccinia striiformis f. sp. tritici
Source: Theor Appl Genet. 2021 Nov 27;135(1):301–19. doi: 10.1007/s00122-021-03967-z (PMC8741662; doi:10.1007/s00122-021-03967-z)
Supplement: Supplementary file 13 — Supplementary file13 (DOCX 4394 kb) [file 122_2021_3967_MOESM13_ESM.docx]

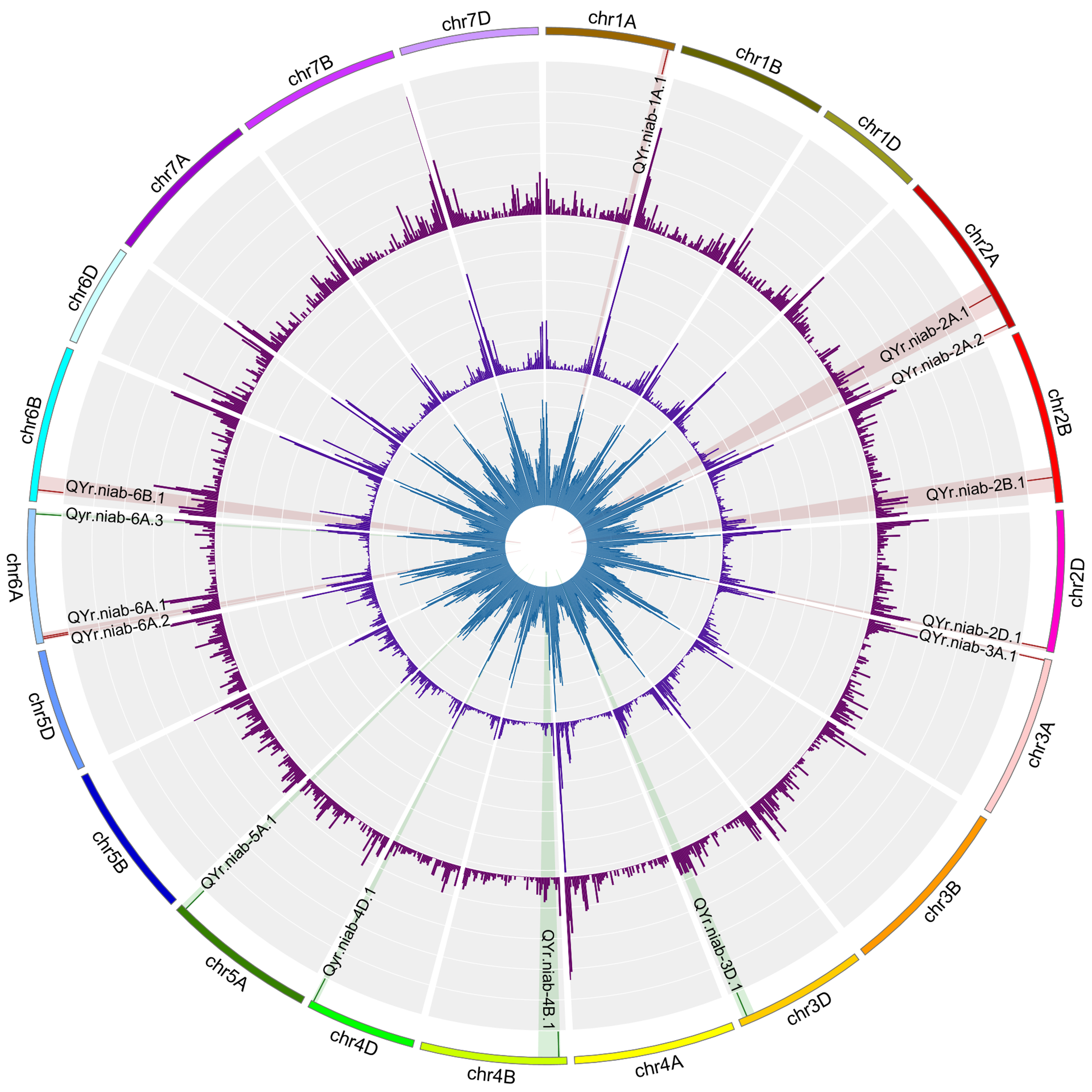


**Supplementary Figure 1.** Circular diagram representing the position of 14 yellow rust (YR) resistance QTL identified in the MAGIC wheat population, in relation to density of candidate ‘resistance genes’ present in the cv. Chinese Spring reference genome (RefSeq v1.0; IWGSC, 2018). Starting from the outermost, Track 1: chromosome number. Track 2: tick marks indicating the position of major (red) and minor (green) YR QTL. Track 3 (purple): histogram of the proportion of resistant genes per gene per 10Mbp. Track 4 (blue): histogram of ‘resistant gene’ count every 10 Mbp. Track 5 (dark blue): histogram of gene count every 10 Mbp.
