# Supplementary material for: Wheat genetic loci conferring resistance to stripe rust in the face of genetically diverse races of the fungus Puccinia striiformis f. sp. tritici
Source: Theor Appl Genet. 2021 Nov 27;135(1):301–19. doi: 10.1007/s00122-021-03967-z (PMC8741662; doi:10.1007/s00122-021-03967-z)
Supplement: Supplementary file 14 — Supplementary file14 (DOCX 48 kb) [file 122_2021_3967_MOESM14_ESM.docx]

**a b**

**c d**

Sus allele

Res allele

c

**Supplementary Figure 2.** Examples of genotyping results for some of the KASP markers developed for the four most significant yellow rust resistance QTL (see Supplementary Table 2 for full details). Genotype calls for resistant (Res) and susceptible (Sus) alleles are indicated, derived from genomic DNA template from the founders subsequently indicated here. NTC = no template negative control. (A) *QYr.niab-1A.1*: KASP marker *tplb0021i12_383* (DNA: Alchemy, susceptible allele; Hereward, resistant allele). (B) *QYr.niab-2A.1*: KASP marker *BS00062679_51* (DNA: Xi19, resistant allele; Claire, susceptible allele). (C) *QYr.niab-2B.1*: KASP marker *BS00016650_51* (DNA: Soissons, resistant allele; Robigus, susceptible allele). (D) *QYr.niab-2D.1*: KASP marker *Kukri_c498_2381* (DNA: Claire, resistant allele; Soissons, susceptible allele).
